# Action-based Modeling of Complex Networks

**DOI:** 10.1038/s41598-017-05444-4

**Published:** 2017-07-27

**Authors:** Viplove Arora, Mario Ventresca

**Affiliations:** School of Industrial Engineering, Purdue University, West Lafayette, IN 47907 USA

## Abstract

Complex networks can model a wide range of complex systems in nature and society, and many algorithms (network generators) capable of synthesizing networks with few and very specific structural characteristics (degree distribution, average path length, etc.) have been developed. However, there remains a significant lack of generators capable of synthesizing networks with strong resemblance to those observed in the real-world, which can subsequently be used as a null model, or to perform tasks such as extrapolation, compression and control. In this paper, a robust new approach we term Action-based Modeling is presented that creates a compact probabilistic model of a given target network, which can then be used to synthesize networks of arbitrary size. Statistical comparison to existing network generators is performed and results show that the performance of our approach is comparable to the current state-of-the-art methods on a variety of network measures, while also yielding easily interpretable generators. Additionally, the action-based approach described herein allows the user to consider an arbitrarily large set of structural characteristics during the generator design process.

## Introduction

Complex networks are used to model real-world systems using sets of nodes and edges that represent elements and their interactions, respectively (the term ‘complex’ refers to any collection of interrelated things in which the pattern of links is meaningful^1^). Complex networks have provided transformative perspectives, models and methods in diverse application domains such as computer science, sociology, chemistry, biology, anthropology, psychology, geography, history and engineering^2^. In particular, network generators are useful for^3, 4^: *simulation* to evaluate sensitivity of network functionality to parameterization, *abnormality detection* for finding subnetworks that are unexpected, *extrapolation* to synthesize larger networks for predicting future network topology, *sampling* to synthesize smaller representative networks to decrease computation time, *compression* to the network generator parameters and obtain an algorithmic description, *control* to influence nodes to achieve a desired outcome of the topology, *anonymization* of a private network to synthesize a similar network for public availability, *null*-*modeling* to assess whether certain network properties are expressed and *structural analysis* to reveal characteristics of the system being studied. Despite the successes, there remains a pressing need for more robust network generators capable of synthesizing networks that are statistically representative of real networks.

The roots of network generators can be traced to random graphs^5–9^, which assume a constant number of nodes and uniform probability on the existence of each link in the network. These generators are not capable of consistently reproducing phenomena observed in the real-world. To overcome this limitation, researchers have proposed subsequent methods for synthesizing networks that exhibit a specific subset of (typically 1–3) characteristics but whose parameterization could be adjusted to better reflect a given real-world system. These approaches focus on controlling network growth or permitting non-uniform link existence^10^ and result in generators capable of reproducing behaviors such as “small-worldness”^11–15^ (i.e. a node can reach other nodes in a small number of steps) and a scale-free degree distribution^16–18^ (i.e., the probability *P*(*k*) ≈ *k* ^−α^, for degree k with usually *α* ∈ (2, 3)). Some contemporary variants of random graphs are capable of exactly reproducing arbitrary degree distributions^19–24^. Models with scale-free degree distribution and adjustable clustering coefficient have also been proposed^25^.

A number of machine learning approaches have been proposed as well. The goal is to learn network generator parameterization from given network observation(s) by maximizing the probability of synthesizing networks with similar global characteristics. Some examples include exponential random graphs^26–28^, latent space models^29^, and stochastic Kronecker graphs^4, 30–32^. While successful, each approach is biased by beliefs their human designer had about the nature of observed networks and the manner that real-world networks evolve. That is, while the algorithms have some degree of freedom, they are inherently constrained by an underlying (rigid) algorithm and consequently are only capable of synthesizing particular networks.

Alternative computation models have also been explored. For instance, cellular automata can model network evolution by applying local agent rules derived by observing collective real-world behavior^33–35^. While robust, these local rules can be very tedious and difficult to derive from an observation of network evolution, especially for nontrivial systems. A symbolic regression technique was recently suggested by^36^, but is limited to probabilistic models and does not consider network growth. A geometric interpretation of popularity versus similarity was used in^37^ to capture various features of real-world networks. *dk*-graphs^38^ model networks as random ensembles, where ensemble size is controlled using *dk*-distributions. An optimization-based approach using information theoretic quantifiers to simulate the dynamic of scale-free networks has also been proposed^39^.

The aforementioned generators were devised by a strategy that focuses on developing algorithms capable of replicating a subset (of typically no more than three) network properties. Ideally, a subset of properties would be sufficient to ensure the realism of synthesized networks with respect to the real-world phenomena being modeled. Unfortunately, this set of network properties is unknown. Additionally, designing mechanisms and models that lead to useful synthetic networks is further complicated by the stochastic local interactions and nonlinear behavior inherent in complex systems. Consequently, recent investigations have been proposed to automate the discovery of network generators for arbitrary global characteristics and phenomena^40^. Such techniques hold significant promise due to their ability to circumvent much of the tedium and creative limitations faced by humans when designing a network generator. In particular, the framework of ^40^ utilzes genetic programming (GP) to evolve a plausible algorithmic description of a user-defined target network but suffers from some drawbacks: (a) the evolutionary search is computationally expensive and scales poorly with network size, (b) it can discover complicated generators, and as a result (c) generators can be difficult to analyze mathematically.

In summary, existing network generators have proven useful in situations where it is already possible to synthesize networks with the desired global characteristics. However, a key limitation is that they are all inherently limited by the manner that they control network formation. Hence, new generators must be continuously developed manually in order to keep pace with the demand for network models exhibiting more and different local and global characteristics. Moreover, the process of scouring literature for potentially useful generators, properly configuring them and then deciding the one(s) that best represent the particular phenomena under study is a daunting task. Consequently, there is a need for robust network generators^3, 4, 41–44^.

Our goal is thus to devise a robust algorithmic framework for learning a compressed model of a given target network, and to show that the resulting generator is capable of synthesizing, with high probability, statistically similar networks to the given network. In order to maximize utility, the framework should be robust to the number and type of global network characteristics that are to be modeled, in addition to yielding easily interpretable generators. The computation time required to design the generator must also not be burdensome.

## Action-based Networks

The novel concept presented herein builds upon the assumption that nodes create, rewire or delete edges by probabilistically choosing from a set of actions that give rise to global network structure (a preliminary version can be seen in^45^. In this context, actions are similar to updating rules in cellular automata, whereby simple spatial neighborhood rules are used to evaluate the next state of a cell. Cellular automata are capable of universal computation using these rules, and very simple deterministic systems can create unpredictable complex behavior^46^. Similarly, it is conjectured that by combining simple actions and carefully choosing corresponding probabilities, we can evolve complex systems by simulating their stochastic local interactions.

**Figure 1:**
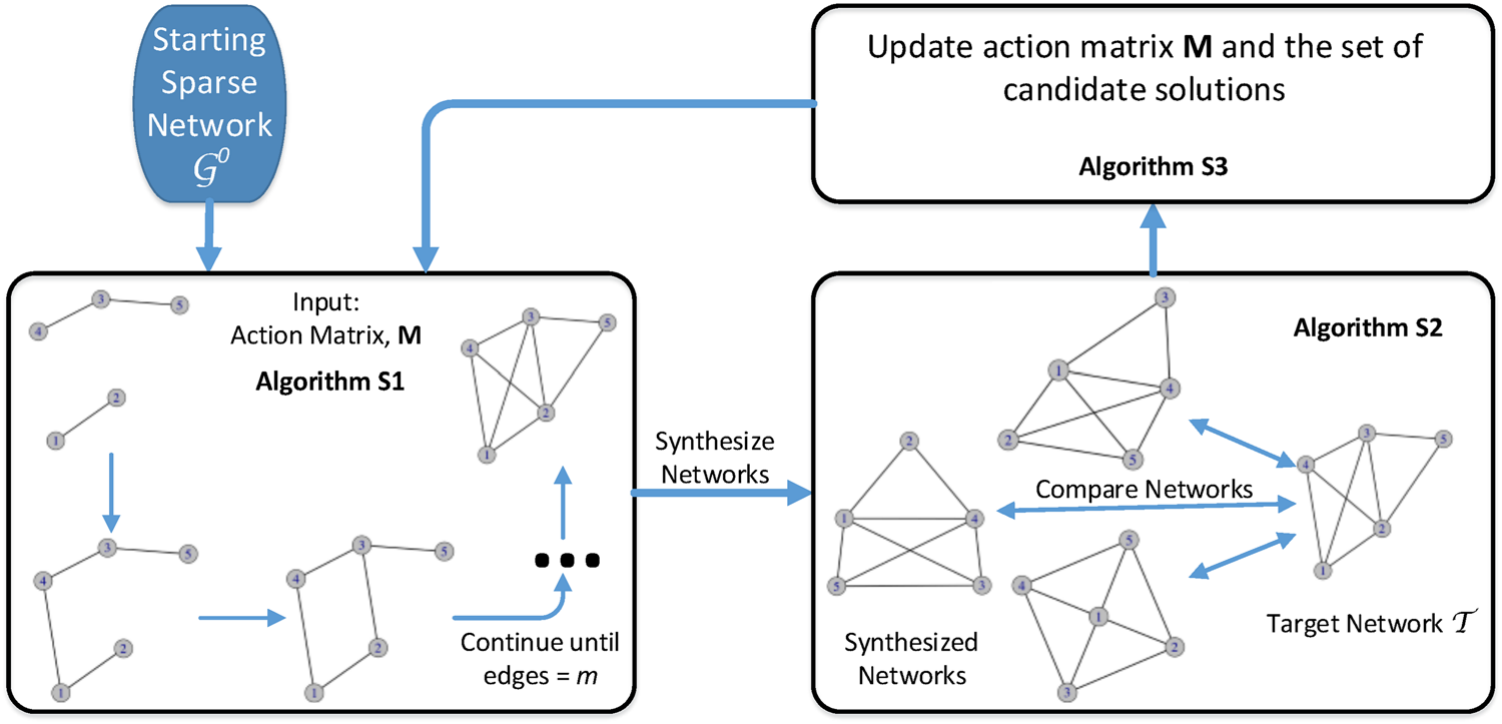
A procedure for determining action matrix **M**: Algorithm S1 probabilistically adds the required number of edges using ABNG-PA(1). Algorithm S2 compares a set of synthesized networks to the target using the user-defined structural characteristics in order to determine the representativeness of action matrix **M**. Algorithm S3 perturbs **M** and retains a set of best-fit solutions. The process repeats until a termination criteria (e.g., number of iterations) is satisfied.

Recent publications^37, 47^ have shed some light on the emergence of power law degree distributions in networks, showing that the interplay between popularity and similarity plays a key role in the organization and evolution of scale-free networks. Building on these results, action-based networks define actions corresponding to different mechanisms of evaluating popularity and (dis)similarity. Actions are used to define local pairwise interaction between nodes to make local topological changes in a network by repeatedly and probabilistically choosing from a pre-defined set of actions, thereby creating a global network structure. A synthesis algorithm *f*(**M**, *n*) can then be used to synthesize networks containing *n* nodes using the learned action-based model **M**, leading to action-based network generators (ABNG). For a given target network, **M** is determined by solving a learning or optimization problem. A pictorial representation of this procedure can be seen in Fig. 1.

More specifically, consider a target network ${\mathscr{J}}=(V,E)$ with *n*_*t*_ = |*V*| nodes and *m*_*t*_ = |*E*| edges. Let the Action Set *A* = {*a*_1_, …, *a*_*k*_} contain *k* node actions. Each action provides a well-defined strategy for selecting the other end *v*_*j*_ of edge (*v*_*i*_, *v*_*j*_), for instance using preferential attachment, node similarity, node dissimilarity, etc. Let *P*_*i*_ be a probability distribution over the *k* actions that can be made by node *v*_*i*_ ∈ *V*. If nodes *v*_*i*′_,*v*_*i*_ ∈ *V* choose actions using the same distribution, there will exist *q* ≤ *n* distinct probability distributions over actions, and each *P*_1_, …, *P*_*q*_ will correspond to a unique *node type*. For ${P}_{q\times k}^{\ast }=[{P}_{i}]$, we define the Action Matrix ${{\bf{M}}}_{q\times (k+\mathrm{1)}}=[{P}^{\ast }|\bar{P}]$, as a condensed representation containing all distinct *P*_*i*_, and probability vector ${\bar{P}}_{q\times 1}$ containing probability of choosing actions according to *P*_*i*_. For a finite set *Y* of user-chosen network characteristics, the problem of determining **M** can be formulated as:

1 $$\begin{array}{lll}{\rm{minimize}} & {\mathbb{E}}[Q({\mathscr{G}}|{\mathscr{J}},Y,{\bf{M}})] & \\ {\rm{subjectto}} & \sum _{j=1}^{k}{M}_{ij}=1 & \forall i=\mathrm{1,}\cdots ,q\\  & \sum _{i=1}^{q}{M}_{ij}=1 & j=k+1\\  & {M}_{ij}\ge 0 & \forall i=\mathrm{1,}\ldots ,q\,{\rm{and}}\,\,\,\forall j=\mathrm{1,}\ldots ,k+1\end{array}$$

where ${\rm{Q}}({\mathscr{G}}|{\mathscr{J}},Y,{\bf{M}})$ is a measure to quantify the dissimilarity between a synthesized network ${\mathscr{G}}=f({\bf{M}},n)$ and target ${\mathscr{J}}$ based on network characteristics *Y.*

### Synthesizing networks

As highlighted in Fig. 1, an algorithm that takes action matrix **M** as input is required to synthesize networks. The principle follows from observations by^48^ who note that there must exist an assembling algorithm to combine various local mechanisms that lead to the emergence of different complex network structures. The action-based framework permits the use of different synthesis algorithms with the options of adding, deleting or rewiring edges (or a combination thereof). Table S1 briefly describes four possible algorithms and their respective complexities for synthesizing a network. More details about these algorithms can be seen in the Supplementary Information Section S3. For the purpose of this paper, we consider a synthesis algorithm that only adds edges to a network and is called ABNG-PA(1).

### Evaluating generator suitability

As seen in Equation (1), a measure *Q* is required to compare a network ${\mathscr{G}}$ synthesized using the generator and the target network ${\mathscr{J}}$. Roots of network comparison can be traced to the graph isomorphism problem^49^, leading to the implicit usage of the notion of network dissimilarity. To maintain the inherent stochastic nature of complex systems, a network generator should ensure that the synthesized networks are not isomorphic. Recent observations have highlighted the need to consider multiple global characteristics when comparing networks^38, 41–44, 50–54^. Ideally, a subset of network properties would be sufficient to capture this dissimilarity, but unfortunately, this set of network properties is unknown (and may not exist). To tackle this problem, ABNG optimizes the action matrix to minimize *Q* for a flexible set of user-defined properties *Y*.

## Results

Six human-designed network generators and 19 real-world networks (see Table S2) were selected to evaluate the efficacy of the action-based approach. Human-devised network generators were selected for historical significance and the distinct global network properties that they model. Another benefit of using these generators is that they are designed to be simple (i.e., nodes use a single strategy for forming edges) and the simplicity should be reflected in their respective action-based models. In all experiments PageRank, degree distribution and betweenness were utilized as the global network characteristics as suggested in^55^, although the approach is indifferent to this choice. Local clustering was added as an objective for networks having more complicated structures. The 2-sample Kolmogorov-Smirnov statistic is used to quantify difference in distribution of these properties between the synthesized and target networks.

### Modeling networks synthesized by human-devised generators

To test the ability of ABNG to replicate distinct global network properties such as scale-free distributions, small world effect etc., the generator was tested using target networks with ≈100 nodes and ≈500 edges synthesized using Erdös-Rėnyi^5^, power law^22^, small world^13^, Barabási–Albert^17^, Forest Fire^56^ and stochastic block models^57^. Having ground-truth network models allows for controlled experimental comparison across network size (number of nodes), as well as direct comparison of the action matrix to the logic of the generator that synthesized the example target network.

Solutions obtained for Erdös-Rėnyi, power law, small world and Barabási–Albert models all resulted in a 1 × (*k* + 1) action matrix **M** ($\bar{P}=1$ in this case). An action matrix corresponding to the solution closest to the origin (based on 1-norm) was chosen as the model for each network, and is shown in Table 1. Only one node type was discovered, implying a homogeneous strategy when forming edges, which is consistent with these network generator algorithms.

**Table 1:** The table shows optimized action matrices for networks synthesized by human-devised generators.

Network↓ | Action→	PAND	PAD	PAPR	PAB	TC	SLW	SJ	NA	$\bar{P}$
Erdös-Rėnyi	0.669	0	0.149	0	0.006	0.007	0.169	0	1
Power Law	0	0.227	0	0.178	0.023	0.076	0	0.496	1
Small World	0.328	0.011	0.023	0	0	0.020	0.618	0	1
Barabási–Albert	0.132	0	0.038	0.560	0.019	0.225	0.026	0	1
Forest Fire	0	0.008	0	0.016	0	0.549	0.051	0.376	0.767
	0	0	0.041	0.547	0.029	0.019	0.216	0.148	0.233
Stochastic Block	0	0.101	0.322	0	0.207	0	0.035	0.335	0.284
	0.090	0.02	0.063	0.102	0.113	0.079	0.363	0.170	0.716

The following actions were used: Preferential attachment on - average neighbor degree (PAND), degree (PAD), PageRank (PAPR) and betweenness (PAB); Triadic closure (TC); Inverse log-weighted (SLW) and Jaccard similarities (SJ); and No action (NA).

**Figure 2:**
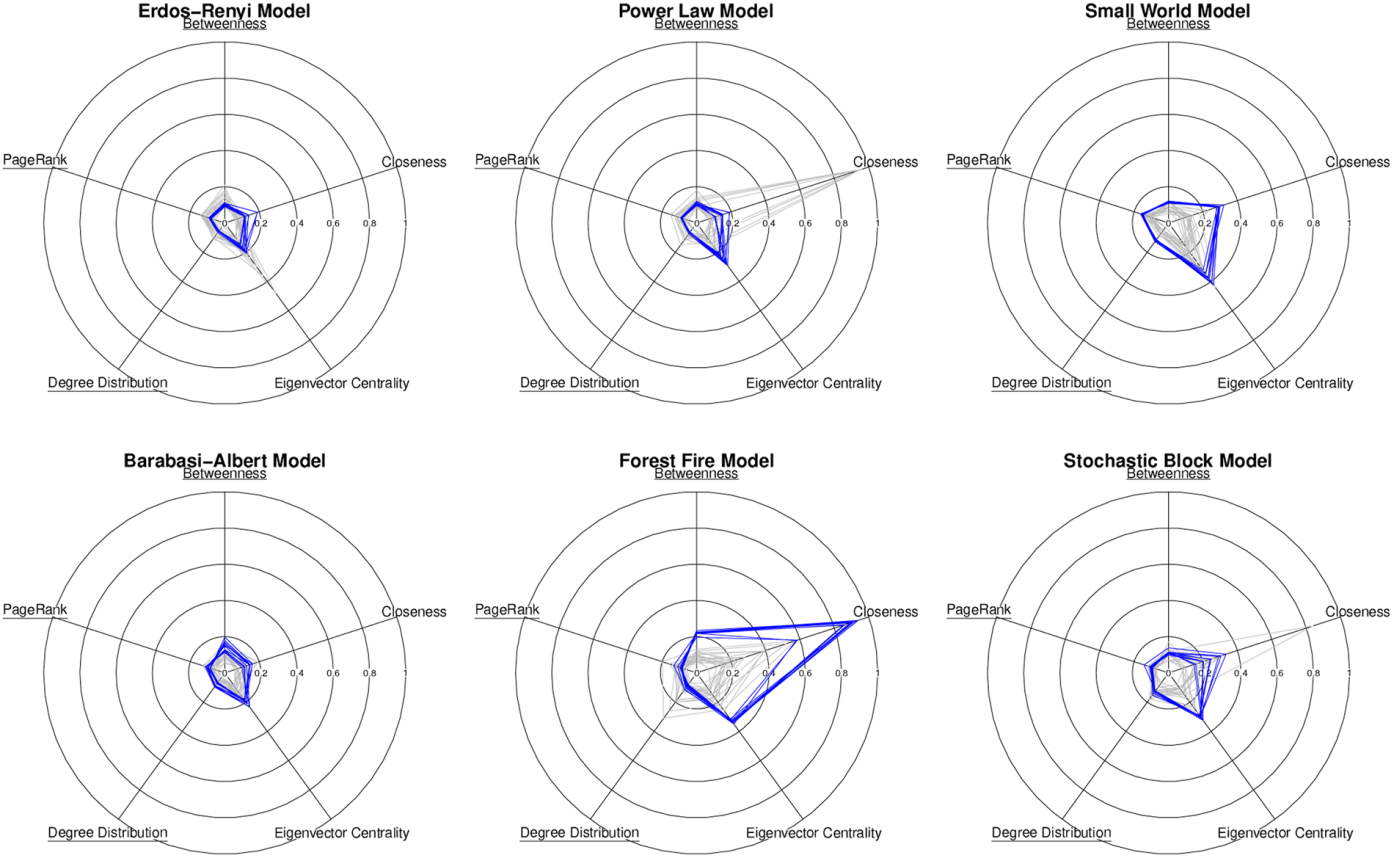
Results obtained from optimized models of human-devised generators. Network properties that were used for optimization are underlined. The plots show KS-test d-statistic with the outer circle showing value of 1 (maximum possible value). The lower the value, better is the synthesized network. Each gray line corresponds to a network synthesized using the target generator. Each blue line corresponds a network synthesized using an optimized action matrix.

The forest fire and block model networks, shown in Fig. 2, required two-row action matrices, indicating the existence of two node types (see Table 1). In Fig. 2, network properties *Y* considered in the optimization process are underlined, others are provided for context and not expected to be near optimal. Lines closer to the origin imply a lower dissimilarity between target and synthesized networks and are thus more desirable. These results suggest that the synthesis algorithm ABNG-PA(1) is capable of modeling the actual network generator using only one target network observation.

**Figure 3:**
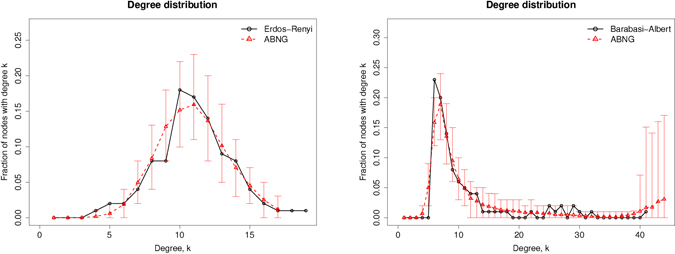
Comparing degree distributions of networks synthesized using ABNG with the target network. The deviation bars capture the range of 90% of the synthesized networks, while the line for ABNG is the mean of 100 synthesized networks.

Figure 3 compares the degree distribution of networks synthesized using ABNG with Erdös-Rėnyi and Barabási–Albert as target networks. The distributions labeled as ABNG are the averaged statistics of 100 synthesized networks. Standard deviation bars capture the range of 90% of the synthesized networks and show that the average degree in the synthesized networks is representative of the target network.

A particularly interesting outcome was obtained when considering the target network synthesized using the stochastic block model generator. In this instance, the target network contained two communities (30 and 70 nodes, respectively) and the resulting 2-row action matrix had ${\bar{P}}_{1}\approx 0.7$ and ${\bar{P}}_{2}\approx 0.3$ (the corresponding action matrix is shown in Table 1). Thus, ABNG was able to accurately infer the existence of two communities and their approximate size in the network. The ability to detect communities in a network is an interesting observation requiring further analysis.

#### Predicting network growth

In order to determine whether a true network generator can be discovered, an experiment is conducted where a known generator synthesizes a network and multiple network snapshots during its growth are recorded. One of the snapshots is selected as a target network for ABNG, and the goal is to ascertain whether all snapshots can be accurately synthesized using the action-based model for the target. Certain assumptions were made for the target network growth: (i) the networks grow linearly with time such that nodes are added one at a time, and (ii) the network is constructed using a consistent strategy where the action matrix is static. In order to accomplish this an action matrix is used to synthesize a network ${{\mathscr{G}}}_{t}$ at time t as learned from the target model, and then the synthesis algorithm iterates to predict the structure of graph ${{\mathscr{G}}}_{t+{t}_{1}}$ and ${{\mathscr{G}}}_{t-{t}_{2}}$ at time *t* + *t*_1_ and *t* − *t*_2_, respectively. If effective, this provides evidence that ABNG-PA(1) can be utilized to predict past or future structures of growing real-world networks only using the action matrix obtained from the present network.

**Figure 4:**
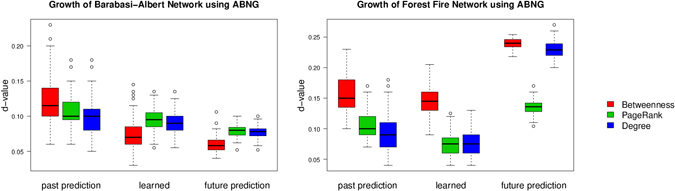
Comparison of results between the actual and the inferred networks. KS-test d-values used for optimization are shown on the y-axis. Line in the middle of each box is for the median value, points show outliers and whiskers represent minimum and maximum values. Similar median d-values (and associated variation) for various network properties of different networks shows that predicted networks are statistically representative of the target networks.

Target networks were synthesized using the Barabási–Albert and forest fire models because they grow networks by adding nodes in a manner that satisfies the aforementioned assumptions. In both cases, a target network was grown to *t* = 500 (i.e., *n* = 500) where snapshots of the network at *t* = 100 and *t* = 200 were recorded. The target network corresponded to the snapshot at *t* = 200 and the resulting optimized action matrix was used to synthesize networks at *t* = 100 and *t* = 500, respectively. Figure 4 provides a comparison between synthesized networks in the three temporal circumstances. The variation and mean of the predicted networks for different network statistics is statistically similar to the learned network. Each plot was generated from 100 synthesized networks.

### Modeling real world networks

**Figure 5:**
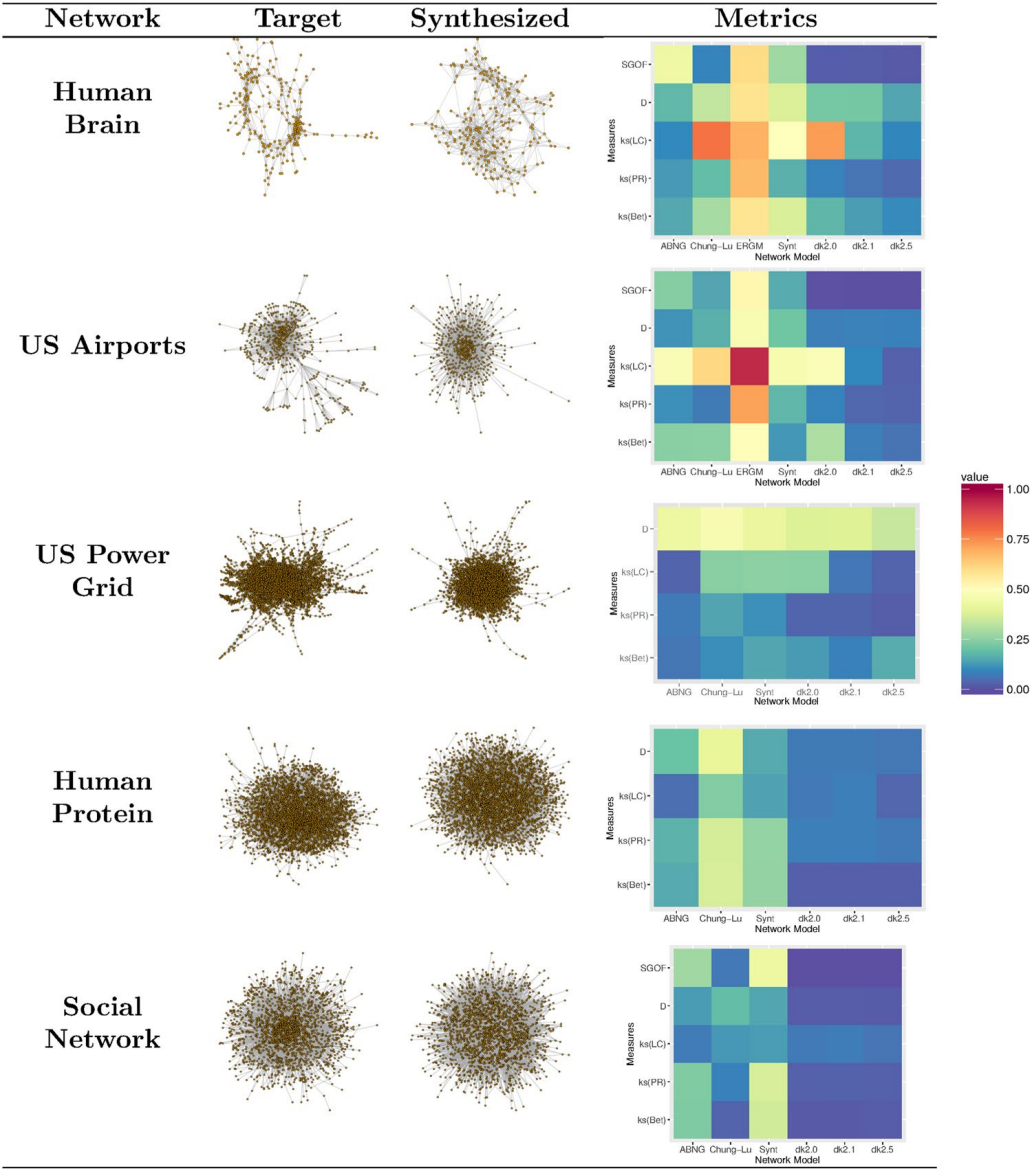
Overview of results for five real-world networks: A visualization of the target network together with the network synthesized using ABNG. Each generator synthesizes 100 networks, and the mean dissimilarity values are recorded in the heat maps. The lower the value, better is the synthesized network.

Complex systems observed in the real-world typically do not have corresponding complex networks that are well modeled by existing standard network generators. Figure 5 presents a summary of the results, featuring heat maps for five of these networks (other results can be found in Supplementary Information Section S7.2), as well as a visual comparison of the target network with the network synthesized using ABNG. Four popular network models, namely Chung-Lu^23, 24^, ERGM^28^, synthetic network generator (Synt)^36^ and dk-random graphs^38^ whose parameters were best fit to the respective target networks to ensure fair comparison are also included in the heat maps. The comparison is conducted based on 5 metrics of dissimilarity, namely: 2-sample Kolmogorov-Smirnov distance based on betweenness, PageRank and local clustering; D-measure introduced in^54^; and the spectral measure of^58^ (this measure was rescaled to lie between 0 and 1 as explained in Supplementary Information Section S7.5). The generators synthesize 100 networks, and the mean values are recorded in the heat maps. Further experimental results are outlined in Supplementary Information Section S7.2. Note that ERGM is not used in the comparison for the US power grid, protein and social networks because it produced errors while synthesizing networks for these cases. Also, the spectral measure is not used for the US power grid and protein networks because of high computation time.

Heat maps of Fig. 5 show that dk-random graphs are the best generator on all measures for each network except power grid, where ABNG is better on two properties. ABNG provides a competitive alternative to dk-random graphs by consistently synthesizing networks having lower or equal dissimilarity to the target, when compared with other generators and some dk-random graph variants.

An action matrix corresponding to the solution closest to the origin (based on 1-norm) obtained for each of these five real-world networks is shown in Table 2. This can help the user in making some conclusions about the structure of these networks. A common observation is that “no action” tends to have high probability for real-world networks. A possible conclusion here is that only a few nodes add edges in a time step and lead to a power law degree distribution in the network (it can be seen in Table 1 that among the human-devised generators, power law network had a high probability for “no action”). For the five networks considered here, we can draw the following conclusions:

- **Human Brain**: Triadic closure and selective triadic closure are the most dominant actions. This is expected as brain networks tend to have very high clustering coefficients^59^. Although triadic closure is the dominant action, some nodes (corresponding to first row of action matrix shown in Table 2) show preferential attachment mechanisms also. This implies that regions of the brain interact with regions having some structural similarity or those that are highly connected.
- **US Airports**: The action-based model obtained has two types of nodes using completely different strategies. The nodes in the first category prefer not to form any connections, while those in the second category connect to nodes based on betweenness i.e. they are more likely to connect to nodes that lie in shortest paths. In the context of airport connectivity, there is a high probability of connecting to “hub” airports.
- **US Power Grid**: The action-based model obtained for the power grid network highlights connection prefernce to “important” nodes specifically based on PageRank, i.e. with nodes of higher quality and quantity of links.
- **Human Protein**: The action-based model for protein interaction shows existence of two types of nodes, one using “no action” with high probability and the other that use preferential attachment mechanisms based on degree and betweenness. Loosely speaking, proteins either prefer not to interact, or otherwise interact with popular nodes with higher probability.
- **Social Network**: Again, the action-based model shows existence of two types of nodes, one using “no action” with high probability and the other that use preferential attachment mechanism based on betweenness. Interestingly, the two node types exist in equal proportions in this network. This leads us to the conclusion that people either tend to interact with popular individuals or not interact at all in this social network.

**Table 2:** The table shows optimized action matrices for five different real-world networks.

Network↓ | Action→	PAND	PAD	PAPR	PAB	TC	SLW	SJ	NA	$\bar{P}$
Human Brain	0	0.016	0.337	0.043	0.195	0.213	0.127	0.069	0.215
	0	0	0	0	0	0.044	0.378	0.578	0.785
US Airports	0	0	0	0.061	0	0.046	0	0.893	0.804
	0	0.013	0.008	0.956	0.001	0	0	0.022	0.196
US Power Grid	0	0	0.597	0.182	0	0.005	0.146	0.070	1
Human Protein	0	0	0	0.282	0	0.009	0.002	0.707	0.723
	0	0.255	0.004	0.724	0.008	0.009	0	0	0.277
Social Network	0	0	0	0.331	0	0	0	0.669	0.516
	0	0.142	0.036	0.803	0	0.015	0	0.004	0.484

The following actions were used: Preferential attachment on - average neighbor degree (PAND), degree (PAD), PageRank (PAPR) and betweenness (PAB); Triadic closure (TC); Inverse log-weighted (SLW) and Jaccard similarities (SJ); and No action (NA).

## Discussion

Action-based network generators provide a flexible framework for reproducing complex structure of networks exhibiting different global/structural statistics by formulating network generation as an optimization problem. The approach consists of three distinct parts: (i) network synthesis using algorithm *f*( · ), (ii) computation of dissimilarity using a user-defined set of measures *Y*, and (iii) an optimization technique to learn parameters **M** for a given target network. Experiments have provided evidence that ABNG can capture different network structural properties observed in a wide variety of networks, but further experimentation is required in the context of networks having various types of community structure. Improved approaches at one or more parts of the action-based framework may provide effective solutions to these challenges.

The current synthesis algorithm uses the coarsest network characteristic, the average degree, leading to a search over a large set of networks. This can be modified by adding additional constraints of synthesizing networks having the same degree sequence^60^ or joint degree distribution^61^. dk-random graphs^38, 54^ have shown that adding these local constraints enhances the capability of a generator to capture the target network. This can also be observed from the low dissimilarity values of the Chung-Lu model for the Social Network, where the synthesized networks were able to reproduce different global characteristics by just matching the expected degree. This coincides with observations of ^38^ that global network properties of the synthesized networks are consequences of copying only a few specific characteristics of the target. Further, it is plausible that many network characteristics are generally correlated and network structure itself may bring out or obfuscate specific correlated characteristics, even if the correlated characteristics aren’t specified as part of the original algorithm goal. The ability to capture certain characteristics might also be completely coincidental as a result of the target network structure.

It should be noted that local clustering corresponds to the 3k-constraint for dk-random graphs, and adding it as an objective provides a way to circumvent the issue of non-existence of sampling from 3k-random graphs. Consequently, adding local clustering to the set of network properties *Y* leads to discovery of better action-based models for real-world networks. Similarly, adding modularity as an objective could lead to preservation of cluster organization in networks with community structure. Other researchers^54^ have shown the existence of tree-like structures in the Power Grid network and used specialized generators to model this network. Figure 5 shows that ABNG and dk-random random graphs don’t adjust well for such networks (especially on the D-measure), but adding specialized actions can provide a potential solution to this problem, although it comes at the extra cost of optimizing the action matrix. Finally, using more sophisticated optimization or likelihood estimation techniques for discovering the action matrix can improve the ability of the ABNG framework to reproduce networks similar to the target.

A strength of the action-based approach lies in its ability to provide a compact representation for networks having many nodes, and can yield insights into the structure of these networks. Preliminary experiments in Supplementary Information Section S7.3 discuss the scaling of ABNG-PA(1) synthesis algorithm with the number of nodes. Practically, ABNG is an appealing option when the user desires to perform tasks such as compression and extrapolation. It should be noted that the scalability of the optimization of **M** depends on the computational requirements of the optimization algorithm, action set, and objectives. ABNG can be particularly useful if the user has some domain specific knowledge about potential actions or network properties for the target network under consideration.

An important factor influencing the capability of ABNG is the choice of actions. In this paper, actions belonging to four different categories were used (preferential attachment, triadic closure, similarity and no action), but utility of actions based on disassortativity or using node features in annotated networks needs to be explored in future research. In general, any local non-random strategy is a potential candidate for an action. In specific contexts, domain specific information can be used (e.g., to ensure constraints on potential node pairings). Existence of a compact set of actions capturing various mechanisms of local interactions among nodes is central to the action-based approach and can potentially provide crucial answers to the relationship between structure, function and dynamics of real-world networks.

## Methods

As shown in Fig. 1, the action-based approach is composed of three algorithms, with the tasks of (1) network synthesis, (2) comparison to target network and (3) optimization of the action matrix. The algorithms used in the current implementation are briefly described here. ABNG-PA(1) is used for network synthesis and it is outlined in detail using Algorithm S1, but briefly the procedure can be described as:

1. Given target network ${\mathscr{J}}=(V,E)$, create initial sparse network ${\mathscr{G}}=(V^{\prime} ,E^{\prime} )$ where |*V*′| = |*V*| and $|E^{\prime} |\ll |E|$
2. Using $\bar{P}$, probabilistically assign a node type to each *v*_*i*_
3. Probabilistically select an action a_l_ for each *v*_*i*_ using *P*_*i*_ ∈ *P* ^*^
4. For each *v*_*i*_, add edge (*v*_*i*_, *v*_*j*_) to ${\mathscr{G}}$ as determined by *a*_*l*_
5. Repeat Steps 3 and 4 until |*E*′| = |*E*|

In this implementation, a starting sparse network is required to synthesize networks using ABNG-PA(1) because some actions can potentially become undefined due of lack of any network characteristics. We create this by randomly sampling 0.7 × *n*_*t*_ edges from ${\mathscr{J}}$ (see Supplementary Information Section S7.4 for experiments on this). Alternative approaches to synthesizing networks from **M** may further improve observed results by allowing deletion and rewiring of edges and therefore capturing different types of local interactions between nodes or allowing construction from an empty starting network.

A multi-objective approach was utilized to evaluate the ability of ABNG to accurately model the desired characteristics of the target network. Let *Y* = {*Y*_1_, *Y*_2_, $\cdots$, *Y*_*N*_} be a set of scale-independent global network properties of interest. For each synthesized network, a 2-sample Kolmogorov-Smirnov statistic is used for each *Y*_*i*_ to quantify difference in distribution from the target, although alternative approaches such as KL-divergence or entropy-related measures are possible. More specifically, the *d* statistic for each pair of synthesized and target networks can be straightforwardly calculated and the mean used as an approximation for the objective function *Y*_*i*_. Further details are outlined in Algorithm S2.

To quantify network structural dissimilarity, the GP system developed in^40^ was used in a meta-analysis in^52^ to evaluate six network centrality measures. Results indicated that of the examined centrality measures, the degree distribution, betweenness centrality, and PageRank were the most effective for quantifying the (dis)similarity between the target and the synthesized networks. We use these three measures; however, the framework allows for any user-desired measures.

Given a synthesis algorithm *f*( · ) and set *Y*, the next goal is to estimate an action matrix based on its average case performance as defined by the optimization problem in Equation (1). To solve this multi-objective search problem, we implement Pareto Simulated Annealing (PSA)^62^, as it is known to be a useful metaheuristic capable of global optimization in a large search space in a fixed amount of time. Additionally, only one evaluation of the objective function is required at each iteration when compared with population-based algorithms, which require an evaluation for each member of the population.

The implementation of ABNG begins with the assumption that each node has the same probability distribution over actions. In other words, we assume that all nodes are initially homogeneous with respect to their preference over actions. This implies that all rows in **P** are identical and the action matrix has dimensions 1 × (*k* + 1) ($\bar{P}=1$ in this case). Additional rows are dynamically added to **M** during the optimization as discussed in Supplementary Information Section S6. The PSA approach explores the solution space by increasing (or decreasing) randomly chosen individual elements of the action matrix, while accepting worse solutions with a probability decreasing exponentially with the number of iterations.

## Electronic supplementary material

Supplementary Information

## References

[1] Newman, M. *Networks*: *An Introduction* (Oxford University Press, 2010).

[2] Bilgin, C. C. & Yener, B. Dynamic Network Evolution: Models, Clustering, Anomaly Detection. *IEEE Networks* (2006).

[3] Chakrabarti, D. & Faloutsos, C. Graph Mining: Laws, Generators, and Algorithms. *ACM Computing Surveys* **38** (2006).

[4] Leskovec J, Chakrabarti D, Kleinberg J, Faloutsos C, Ghahramani Z (2010). Kronecker graphs: An approach to modeling networks. The Journal of Machine Learning Research.

[5] Erdös P, Rényi A (1959). On random graphs. Publicationes Mathematicae Debrecen.

[6] Erdös, P. & Rényi, A. On the Evolution of Random Graphs. In *Publication of the mathematical institute of the hungarian academy of sciences* 17–61 (1960).

[7] Gilbert EN (1959). Random Graphs. The Annals of Mathematical Statistics.

[8] Solomonoff R, Rapoport A (1951). Connectivity of Random Nets. Bulletin of Mathematical Biology.

[9] Bollobás, B. *Random Graphs* (Academic Press, 1985).

[10] Fienberg SE (2012). A Brief History of Statistical Models for Network Analysis and Open Challenges. Journal of Computational and Graphical Statistics.

[11] Milgram S (1967). The Small World Problem. Psychology Today.

[12] Travers J, Milgram S (1969). An Experimental Study of the Small World Problem. Sociometry.

[13] Watts, D. J. & Strogatz, S. H. Collective dynamics of’small-world’ networks. *Nature* 440–442 (1998). 10.1038/30918 9623998

[14] Barrat A, Weigt M (2000). On the properties of small-world network models. The European Physical Journal B.

[15] Kleinberg, J. The Small-world Phenomenon: An Algorithmic Perspective. In *Thirty*-*second Annual ACM Symposium on Theory of Computing* 163–170 (2000).

[16] Bollobás B, Riordan O, Spencer J, Tusnády GE (2001). The degree sequence of a scale-free random graph process. Random Structures and Algorithms.

[17] Barabasi A-L, Albert R (1999). Emergence of scaling in random networks. Science.

[18] Esquivel-Gomez J, Stevens-Navarro E, Pineda-Rico U, Acosta-Elias J (2015). A growth model for directed complex networks with power-law shape in the out-degree distribution. Nature Scientific Reports.

[19] Boguñá M, Pastor-Satorras R (2003). Class of correlated random networks with hidden variables. Physical Review E.

[20] Bender EA, Canfield ER (1978). The asymptotic number of labeled graphs with given degree sequences. Journal of Combinatorial Theory, Series A.

[21] Wormald, N. C. *Some Problems in the Enumeration of Labelled Graphs*. Ph.D. thesis, University of Newcastl (1978).

[22] Aiello W, Graham FC, Lu L (2001). A Random Graph Model for Power Law Graphs. Experimental Mathematics.

[23] Chung F, Lu L (2002). The average distances in random graphs with given expected degrees. Proceedings of the National Academy of Sciences of the United States of America.

[24] Chung F, Lu L (2002). Connected Components in Random Graphs with Given Expected Degree Sequences. Annals of Combinatorics.

[25] Herrera, C. & Zufiria, P. J. Generating Scale-free Networks with Adjustable Clustering Coefficient Via Random Walks 1–6 (2011).

[26] Strauss D (1986). On a General Class of Models for Interaction. SIAM Review.

[27] Wasserman S, Pattison P (1996). Logit models and logistic regressions for social networks. Psychometrika.

[28] Anderson CJ, Wasserman S, Crouch B (1999). A p* primer: logit models for social networks. Social Networks.

[29] Hoff PD, Raftery AE, Handcock MS (2002). Latent space approaches to social network analysis. Journal of the American Statistical Association.

[30] Leskovec, J., Chakrabarti, D., Kleinberg, J. & Faloutsos, C. Realistic, Mathematically Tractable Graph Generation and Evolution, Using Kronecker Multiplication. In *9th European Conference on Principles and Practice of Knowledge Discovery in Databases* 133–145 (2005).

[31] Leskovec, J., Kleinberg, J. & Faloutsos, C. Graphs over Time: Densification Laws, Shrinking Diameters and Possible Explanations. In *Eleventh ACM SIGKDD International Conference on Knowledge Discovery in Data Mining* 177–187 (2005).

[32] Leskovec, J. & Faloutsos, C. Scalable Modeling of Real Graphs Using Kronecker Multiplication. In *24th International Conference on Machine Learning* 497–504 (2007).

[33] Lukeman R, Li Y-X, Edelstein-Keshet L (2010). Inferring individual rules from collective behavior. Proceedings of the National Academy of Sciences.

[34] Kayama, Y. Complex networks derived from cellular automata. *arXiv*:*1009*.*4509* (2010).

[35] Yang, X. S. & Yang, Y. Z. L. Cellular automata networks. In *Proceedings of Unconventional Computing* 280–302 (2007).

[36] Menezes T, Roth C (2014). Symbolic regression of generative network models. Scientific Reports.

[37] Papadopoulos F, Kitsak M, Serrano MA, Boguna M, Krioukov D (2012). Popularity versus similarity in growing networks. Nature.

[38] Orsini C (2015). Quantifying randomness in real networks. Nature Communications.

[39] Schieber, T. A. & Ravetti, M. G. Simulating the dynamics of scale-free networks via optimization. *PLoS ONE* **8** (2013). 10.1371/journal.pone.0080783 PMC3865918 24353752

[40] Bailey A, Ventresca M, Ombuki-Berman B (2014). Genetic Programming for the Automatic Inference of Graph Models for Complex Networks. IEEE Transactions on Evolutionary Computation.

[41] Nataša P (2007). Biological network comparison using graphlet degree distribution. Bioinformatics.

[42] Bigdeli, A., Tizghadam, A. & Leon-Garcia, A. Comparison of Network Criticality, Algebraic Connectivity, and Other Graph Metrics. In *1st Annual Workshop on Simplifying Complex Network for Practitioners*, 4:1–4:6 (ACM, 2009).

[43] Roy S (2012). Systems biology beyond degree, hubs and scale-free networks: the case for multiple metrics in complex networks. Systems and Synthetic Biology.

[44] Yaveroglu ÖN (2014). Revealing the Hidden Language of Complex Networks. Scientific Reports.

[45] Arora V, Ventresca M (2016). A Multi-objective Optimization Approach for Generating Complex Networks. Proceedings of the 2016 on Genetic and Evolutionary Computation Conference Companion - GECCO ’16 Companion.

[46] Mitchell, M. *Complexity*: *A guided tour* (Oxford University Press, 2009).

[47] Barabási A-L (2012). Network science: Luck or reason. Nature.

[48] Zheng B (2014). A simple model clarifies the complicated relationships of complex networks. Sci Rep.

[49] Luks EM (1982). Isomorphism of graphs of bounded valence can be tested in polynomial time. Journal of Computer and System Sciences.

[50] Fay, D., Moore, A. W., Brown, K., Filosi, M. & Jurman, G. Graph metrics as summary statistics for Approximate Bayesian Computation with application to network model parameter estimation. *Journal of Complex Networks* cnu009 (2014).

[51] Goni J (2014). Resting-brain functional connectivity predicted by analytic measures of network communication. Proceedings of the National Academy of Sciences.

[52] Harrison, K. R., Ventresca, M. & Ombuki-Berman, B. Investigating Fitness Measures for the Automatic Construction of Graph Models. In (eds) Mora, A. M. & Squillero, G. *EvoApplications*, vol. 9028 of *Lecture Notes in Computer Science* 189–200 (Springer, 2015).

[53] Habibi J, Movaghar A, Rashidian S, Aliakbary S, Motallebi S (2015). Distance metric learning for complex networks: towards size-independent comparison of network structures. Chaos: An Interdisciplinary Journal of Nonlinear Science.

[54] Schieber TA (2017). Quantification of network structural dissimilarities. Nature Communications.

[55] Harrison, K. R., Ventresca, M. & Ombuki-Berman, B. M. A meta-analysis of centrality measures for comparing and generating complex network models. *Journal of Computational Science* (in press) (2016).

[56] Leskovec, J., Kleinberg, J. & Faloutsos, C. Graph Evolution: Densification and Shrinking Diameters. {*ACM*} *Transactions on Knowledge Discovery from Data* **1** (2007).

[57] Faust K, Wasserman S (1992). Blockmodels: Interpretation and evaluation. Social Networks.

[58] Shore J, Lubin B (2015). Spectral goodness of fit for network models. Social Networks.

[59] Bullmore E, Sporns O (2009). Complex brain networks: graph theoretical analysis of structural and functional systems. Nature Reviews Neuroscience.

[60] Molloy, M., Reed, B., Combinatoire, E. & Pierre, U. The size of the giant component of a random graph with a given degree sequence 1 Introduction and Overview 1–16 (2000).

[61] Stanton I, Pinar A (2012). Constructing and sampling graphs with a prescribed joint degree distribution. Journal of Experimental Algorithmics.

[62] Czyzak P, Jaszkiewicz A (1998). Pareto Simulated Annealing–A Metaheuristic Technique for Multiple-Objective Combinatorial Optimization. Journal of Multi-Criteria Decision Analysis.

