# Supplementary material for: Action-based Modeling of Complex Networks
Source: Sci Rep. 2017 Jul 27;7:6673. doi: 10.1038/s41598-017-05444-4 (PMC5532273; doi:10.1038/s41598-017-05444-4)
Supplement: Supplementary file 1 — Supplementary Information [file 41598_2017_5444_MOESM1_ESM.pdf]

Supplementary Information for "Action-based Modeling of Complex Networks"

Viplove Arora
arora34@purdue.edu

Mario Ventresca
mventresca@purdue.edu

May 22, 2017

S1 Introduction

In this Supplementary Information, we first introduce a general form of the network generation problem in Section S2, which discusses how the problem of network generation can be turned into an optimization problem and how the results can be interpreted. Section S3 outlines details regarding alternative synthesis algorithms and also briefly discusses computational complexity of the different synthesis algorithms. Section S5 provides an algorithmic description for the action-based synthesis algorithm and network comparison techniques used in the implementation discussed in this paper. This is followed by a small example to illustrate the working of the current ABNG synthesis algorithm in Section S4. Section S6 discusses an implementation of the action-based approach under some simplifying assumptions, followed by how an optimization algorithm can be used to discover an action-based model for a given target network. Finally, we present extended results in Section S7 and also provide details about the relevant packages and methods used to extract networks from data-sets.

S2 The Network Generation Problem

A common theme in various network generating models is estimating the likelihood $P(\mathcal{G}|\mathcal{T})$ of a synthesized network $\mathcal{G}$ under some model, which involves comparing the synthesized network(s) to the target $\mathcal{T}$. Ultimately, the goal is to estimate network model parameters that maximize the algorithm's ability to synthesize networks matching the desired structural properties of $\mathcal{T}$. To achieve this, we first need to define a set of network properties ($Y = \{Y_1, \cdots, Y_k\}$) to be matched, then define a quality of fit $Q(\mathcal{G}|\mathcal{T}, Y, X)$ to quantify (dis)similarity and finally optimize the generator parameters $X = \{x_1, \cdots, x_j\}$ over the feasible domain D.

This can be written into the following optimization problem:

$$\begin{aligned} &\text{maximize} \quad \mathbb{E}\left[Q(\mathcal{G}|\mathcal{T}, Y, X)\right] \\ &\text{subject to} \quad X \in D \end{aligned} \tag{S1}$$

Here, we assume that maximizing the $\mathbb{E}\left[Q(\mathcal{G}|\mathcal{T}, Y, X)\right]$ (the expectation is taken for the set of synthesized networks to account for stochastic variations in the network generator) is equivalent to maximizing the likelihood $P(\mathcal{G}|\mathcal{T})$.

A network comparison technique evaluates $Q(\mathcal{G}|\mathcal{T}, Y, X)$, and it should ensure that the difference between $\mathcal{G}$ and $\mathcal{T}$ should not exceed what is expected from mere population variability or stochastic fluctuations [3]. The set of network properties Y consists of global measures that contain information about the topological characteristics of a network. Matching these global properties can synthesize networks that are representative of the entire target network structure as compared to small groups of or individual nodes.

Let us define a threshold θ that defines the range in which $\mathbb{E}\left[Q(\mathcal{G}|\mathcal{T}, Y, X)\right]$ becomes *acceptable* and can be used to say that the networks $\mathcal{G}$ and $\mathcal{T}$ are *sufficiently similar*. This implies that if $\theta = 0$, the problem reduces to exactly matching all possible target network statistics, and hence the optimal solution would be an isomorphic graph. The network generation problem is not the same as graph isomorphism, as the goal here is not to exactly reproduce the target network but rather learn a model that can synthesize networks statistically similar to one another and the target network i.e. variation is expected/desired. So, setting an acceptable threshold θ on each objective function (global network statistic) can help us find *good* generators for a target network. Also, because network generators are stochastic algorithms, the networks synthesized by fixing the parameters of a model will not be isomorphic to each other or the target network.

S3 Synthesis Algorithms

As highlighted in Section 2 and Figure 1, a synthesis algorithm is required to synthesize networks using the action-based approach. A synthesis algorithm $f(\mathbf{M}, \cdot)$ uses an action matrix $\mathbf{M}$ and the local interaction mechanism of actions to synthesize networks. The action-based framework permits the use of different synthesis algorithms with the options of adding, deleting or rewiring edges (or a combination thereof). Table S1 briefly describes four possible algorithms and their respective complexities for synthesizing a network. The complexity of each algorithm is given in terms of action calls, which gives the expected number of times an action will be evaluated during network synthesis. Detailed description of each algorithm is given below:

- **ABNG-PA(·):** This synthesis algorithm corresponds to addition of edges to a starting network. It follows from preferential attachment algorithms, where new edges are added in each time step. The parameter in ABNG-PA(·) denotes the number of action-based queries to each node for addition of an edge. The current implementation uses ABNG-PA(1) as the synthesis algorithm i.e. each node is queried for addition of a single edge in a time step. Clearly, the computational complexity of this algorithm is proportional to the number of edges that need to be added to the starting network.

- **ABNG-D(·):** This synthesis algorithm modifies ABNG-PA(·) by deleting edges from a starting network having more edges than the target. Again, the input parameter denotes the number of edge deletion queries in each time step during network synthesis. The computational complexity of this algorithm is proportional to the number of edges that need to be deleted from the starting network.
- **ABNG-R:** Another local operation that actions can perform is rewiring i.e. changing one end of an already existing edge. The rewiring technique is used in dk-random graphs [21] to sample from the entire ensemble of networks. In context of ABNG, ABNG-R starts with the original target network and rewires its edges using actions. Every edge of each node is queried for an action-based rewiring resulting in a total of $2m_t$ action calls.
- **ABNG-C:** ABNG-C corresponds to a degree sequence preserving extension of ABNG-PA. It can be seen as a constrained version ABNG-PA where the degree sequence of the target network is preserved. Each node i can add edge (v_i, v_j) under the condition that the degree sequence is not violated. Joint degree distribution preserving synthesis algorithms is also a possibility. It can be seen that starting from a network with no edges, this synthesis algorithm needs to add m_t edges leading to computational complexity directly proportional to m_t.

Table S1: Synthesis algorithms for ABNG: ρ is the probability an edge will be added by a given action matrix, and m_s is the number of edges in the starting network.

Synthesis Algorithm	Description	Action calls
ABNG-PA(·)	Action-based addition of edges to a starting network with $m_s \ll m_t$	$\frac{1}{\rho}(m_t - m_s)$
ABNG-D(·)	Action-based deletion of edges from a starting network with $m_t \ll m_s$	$\frac{1}{\rho}(m_s - m_t)$
ABNG-R	Action based rewiring of all edges in the target network	$2m_t$
ABNG-C	Degree sequence preserving action-based addition of edges to an empty	$\frac{1}{\rho}m_t$

It should be noted that the computational complexity of a synthesis algorithm can be upper-bounded by considering the complexity of most expensive action in the Action set. Also, as highlighted in Table S1, the computational complexity of a synthesis algorithm depends on the action matrix being used as input or ρ, which is the probability an edge will be added by a given action matrix.

S4 A Worked Example

In ABNG, nodes synchronously create edges in discrete time steps. For example, at $t = 0$ we are given a sparse starting network and actions for each node are evaluated based on the network given at $t = 0$, followed by creation of new networks at $t = 1$, which are used for evaluation of edges to be added at $t = 2$ and so on. Also, the new edge is randomly chosen, independently of the others, according to a distribution depending only on a finite set of actions. To better understand the working of the ABNG framework, let us consider a

simple example. Consider a starting network $\mathcal{G}_0$ (shown in Figure S1a) at $t = 0$ with adjacency matrix $\mathbf{A}_0$, an action matrix $\mathbf{M} = [0.7 \quad 0.3]$, and ABNG with two hypothetical actions a_1 and a_2. We compute matrices $\mathbf{A}_0^1$ (for this example, it is assumed that a_1 is an action based on preferential attachment on node degree and is used for generating the matrix $\mathbf{A}_0^1$) and $\mathbf{A}_0^2$, where the ith row of $\mathbf{A}_0^1$ corresponds to $\hat{p}_i$ obtained by using action a_1. The networks synthesized by ABNG (at $t = 1$) can be sampled using $\mathbf{A}_1 = 0.7\mathbf{A}_0^1 + 0.3\mathbf{A}_0^2 + \mathbf{A}_0$, where each element of $\mathbf{A}_1$ corresponds to the probability of existence of an edge. Three networks synthesized using $\mathbf{A}_1$ are shown in S1b. The networks synthesized at $t = 1$ can now be used as starting networks for $t = 2$. We gain several key insights from this example:

- The actions a_1 and a_2 belong to two different categories of actions, namely probabilistic and deterministic. In a deterministic action, a node v_i selects another node v_j to create an edge, whereas in a probabilistic action there exist probabilities of connecting to different nodes.
- For the rows of $\mathbf{A}_0^1$, it can be seen that the sum is < 1. This means it is feasible that a node might not create an edge even after choosing an action.
- It can be clearly seen that the condition for no multi-edges or self loops is enforced. All corresponding entries have zeros in $\mathbf{A}_0^1$ and $\mathbf{A}_0^2$.
- It should be noted that the matrices must remain symmetric in case of undirected networks.

$$\mathbf{A}_0 = \begin{bmatrix} 0 & 1 & 1 & 0 & 0 \\ 1 & 0 & 0 & 0 & 0 \\ 1 & 0 & 0 & 1 & 0 \\ 0 & 0 & 1 & 0 & 0 \\ 0 & 0 & 0 & 0 & 0 \end{bmatrix} \quad \mathbf{A}_0^1 = \begin{bmatrix} 0 & 0.167 & 0.333 & 0.167 & 0 \\ 0.333 & 0 & 0.333 & 0.167 & 0 \\ 0.333 & 0.167 & 0 & 0.167 & 0 \\ 0.333 & 0.167 & 0.333 & 0 & 0 \\ 0.333 & 0.167 & 0.333 & 0.167 & 0 \end{bmatrix} \mathbf{A}_0^2 = \begin{bmatrix} 0 & 0 & 0 & 0 & 1 \\ 0 & 0 & 1 & 0 & 0 \\ 0 & 0 & 0 & 0 & 1 \\ 1 & 0 & 0 & 0 & 0 \\ 0 & 0 & 0 & 0 & 0 \end{bmatrix} \tag{S2}$$

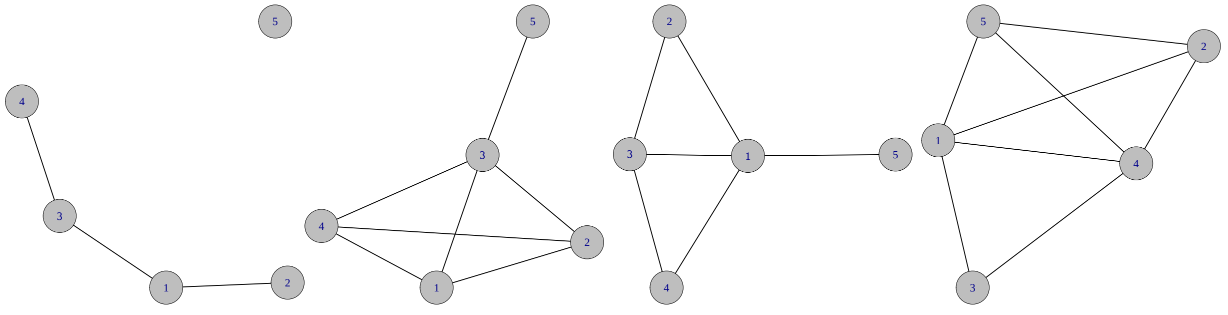

(a) (b)

Figure S1: Network synthesis process of ABNG: S1a The initial starting network $\mathcal{G}_0$ having adjacency matrix $\mathbf{A}_0$. S1b Three networks synthesized at $t = 1$ as explained in the example. It must be noted that the networks shown here have different edges.

S5 Algorithms

Algorithm S1 ABNG

Input: $\mathbf{M}$, a $q \times (k+1)$ matrix satisfying Equation 1, a starting network $\mathcal{G} = (V', E')$, a target network $\mathcal{T} = (V, E)$ and a set A of k actions
while $|E'| < |E|$, for every node v_i in $\mathcal{G}$ **do**
  $P_i = P^*[z, :] \quad wp \quad \bar{P}_z, \quad z \leq q$
  choose a_l for $v_i \quad wp \quad P^*_{il} \quad l = 1, \cdots, k$
  $j = a_l(V|i)$
  $E(\mathcal{G}) = E(\mathcal{G}) \cup (i, j)$
end while
return $\mathcal{G}$

Algorithm S2 Network Comparison

Input: $G = \{\mathcal{G}_1, \cdots, \mathcal{G}_n\}$ is a set of synthesized networks and $\mathcal{T}$ is the target network
for all $\mathcal{G}_j \in G$ **do**
  for all $Y_i(\mathcal{G}_j) \in Y$ **do**
    $d_i = \sup_{x \in \mathbb{R}} |F_{i,\mathcal{T}}(x) - F_{i,\mathcal{G}_j}(x)|$
  end for
end for
return $(\mathbb{E}(d_1), \cdots, \mathbb{E}(d_k))$ ▷ $\mathbb{E}(d)$ is an estimation of the expected value.

S6 Proposed Implementation

In this section, a simple yet effective way of implementing the abstract concept of ABNG discussed in Section 2 is presented. The goal is to compute (estimate) an action matrix that optimizes ABNG based on its *average case performance* as defined by the optimization problem in Equation 1. To solve this multi-objective search problem, we implement Pareto Simulated Annealing (PSA) [11], as it is known to be a useful metaheuristic capable of global optimization in a large search space in a fixed amount of time (or iterations). It is also beneficial due to the fact that only one evaluation of the objective function is required at each iteration when compared with population-based GA algorithms, which require an evaluation for each member of the population.

The GP system developed in [4] was used in a meta-analysis in [14] to evaluate six network centrality measures. Results indicated that of the examined centrality measures, the *degree distribution, betweenness centrality,* and *PageRank* were the most effective for quantifying the (dis)similarity between the target and the synthesized networks. We will use these three measures; however, the framework allows for any user-

desired measures.

The implementation of ABNG starts with the assumption that each node has the same probability distribution over actions. In other words, we assume that all nodes are homogeneous with respect to their preference over actions that in turn influences their choices for creating specific edges in the network. This implies that all rows in $\mathbf{P}$ are identical and the action matrix has dimensions $1 \times (k+1)$. Additional rows are dynamically added to $\mathbf{M}$. This is discussed in detail with the optimization algorithm in this section. The PSA approach explores the solution space by increasing (or decreasing) individual elements (at each iteration, an element is chosen uniformly at random) of the action matrix to find better solutions, while accepting worse solutions with a probability decreasing with the number of iterations.

S6.1 Assumptions

Before going into further details of the optimization algorithm, we list the simplifying assumptions in the current implementation of ABNG:

1. *Input network types:* It is assumed that all of the target and synthesized networks are simple graphs, i.e undirected with no self edges. The networks considered for experiments were also unweighted and unlabelled. This does not imply that ABNG is not applicable to such networks.

2. *Network objectives:* No community-specific objectives were considered, although communities are likely in real-world networks. If needed, special objectives can be added to the optimization framework to synthesize networks with more specific community structure.

3. *Starting network:* The current implementation of ABNG needs a starting network with n nodes as input. Furthermore, this starting network cannot be empty because some actions can potentially become undefined because of lack of any network characteristics. For example, an action based on preferential attachment according to degree of a node would essentially be equivalent to randomly selecting a node in case of an empty network because each node will have a degree of 0. To tackle these issues, we create $\mathcal{G}^0$, a starting sparse network (edges $\ll$ nodes), as input.

4. *Fitness:* As seen in Algorithm S1, even though the network is built over multiple iterations per node, ABNG does not evaluate characteristics for these interim networks. That is, only the target network characteristics are considered by the evaluation function.

5. *Types of actions:* As discussed in Section 5, the synthesis algorithm in the current implementation is restricted to adding edges only. This is a reasonable assumption because we only consider static target networks, which can be synthesized using this restricted subset of actions.

6. *Stopping criteria:* The network synthesis process of Algorithm S1 is terminated when the number of edges in the network being synthesized is equal to the number of edges in the target network.

S6.2 Optimizing the Action Matrix

Algorithm S3 Pareto Simulated Annealing

1: **Input:** $\mathbf{M}_0 \in D : q \times (k+1)$ action matrix
2: $S \leftarrow \emptyset$, $z = 1$
3: **while** $z \leq iter$ **do**
4: $\quad \alpha = e^{-\beta \times z}$ ▷ β is some constant
5: $\quad \mathbf{M} \in B(\mathbf{M}_{z-1})$ ▷ find an action matrix in the neighborhood of the current solution
6: $\quad \mathcal{G} \leftarrow ABNG(\mathbf{M})$ ▷ Algorithm S1
7: $\quad$ **while** $S \not\prec \mathbf{M}$ **do**
8: $\quad\quad S \subseteq S \cup \mathbf{M}$ ▷ update set of efficient solutions
9: $\quad\quad \mathbf{M}_{z-1} \leftarrow \mathbf{M}$
10: $\quad\quad \mathbf{M} \in B(\mathbf{M}_{z-1})$ ▷ update the same element of $\mathbf{M}$ as in line 5
11: $\quad\quad \mathcal{G} \leftarrow ABNG(\mathbf{M})$ ▷ Algorithm S1
12: $\quad$ **end while**
13: $\quad \mathbf{M}_z \leftarrow \mathbf{M} \quad w.p. \quad \alpha$
14: $\quad \mathbf{M}_z \leftarrow \mathbf{M}_{z-1} \quad w.p. \quad 1-\alpha$
15: $\quad S_z \leftarrow S$
16: $\quad$ **if** $z = iter$ & no change in S for 100 iterations **then**
17: $\quad\quad$ **return** S
18: $\quad$ **else**
19: $\quad\quad iter = iter + 100$
20: $\quad$ **end if**
21: $\quad z = z + 1$
22: **end while**
23: **return** $\mathcal{G}$

Pareto Simulated Annealing [11] is the multi-objective analog of simulated annealing. It provides a procedure to search for a set of solutions to a multi-objective combinatorial optimization problem as shown in Algorithm S3 . Due to multiple objectives in the problem formulation, more than one efficient solution can exist. Let D be the set of feasible solutions following the constraints defined in the optimization problem in Equation 1. For a solution $\mathbf{M}$, $B(\mathbf{M}) \subseteq D$ is the neighborhood of solution $\mathbf{M}$ obtained by changing the probability of only one action at a time, i.e. only one element of the matrix $\mathbf{M}$ is increased (or decreased) in a single iteration of PSA (while keeping the solution feasible as defined in the constraints in Equation 1). Our adapted procedure of PSA starts with an action matrix $\mathbf{M}_0$ generated uniformly at random from D to prevent any bias due to a starting point. A new solution $\mathbf{M}_z \in B(\mathbf{M}_{z-1})$ is generated using the procedure shown in Algorithm S3 , where $B(\mathbf{M}_{z-1}) \subseteq D$ is the set of feasible solutions that can be reached from

$\mathbf{M}_{z-1}$ by making a simple move that can only increase (or decrease) the value of one element of $\mathbf{M}_{z-1}$, while maintaining the feasibility of the solution.

Owing to the multi-objective nature of the problem, we need to maintain a set S of potentially efficient solutions. A solution $\mathbf{M} \in D$ is efficient (Pareto-optimal) if there is no $\mathbf{M}' \in D$ such that $\forall j \; Y_j(\mathbf{M}') \leq Y_j(\mathbf{M})$ and $Y_j(\mathbf{M}') < Y_j(\mathbf{M})$ for at least one j. S is updated with $\mathbf{M}_z$ if it is not Pareto dominated [1] by the solutions in S. The algorithm repeatedly increases (or decreases) the value of the same element of $\mathbf{M}_z$ while it continues to be non-dominated by the current set of potentially efficient solutions (Algorithm S3 lines 7-12). This is particularly useful for optimizing the action matrix because it was observed that for the networks considered in this paper, nodes tend to connect based on simple decisions leading to solutions where a subset of actions have high probability, whereas other actions might have zero or near-zero probability. Finally, if $\mathbf{M}_z$ is not added to the Pareto front, the algorithm moves to the new solution with probability α that decays exponentially with the number of iterations, otherwise it returns to the previous solutions $\mathbf{M}_{z-1}$.

The optimization procedure could terminate at a local optima or some other non-optimal stationary point. To help overcome this issue, we use multiple starting points for the optimization process, i.e. the procedure starts with more than one $\mathbf{M}_0$. In the current implementation, we attempt to find the most simple generator (in terms of how nodes make decisions for connecting to other nodes) for the target network. In terms of the action matrix, the simplest generator can be obtained when all nodes have a common mechanism (same probability distribution over actions) for forming edges, and hence $\mathbf{M}$ is assumed to have dimensions $1 \times (k + 1)$. This is followed by dynamically adding more 'node types' (or rows in the action matrix) to the current solutions. In the algorithm implementation, once a Pareto front (or a set of potentially efficient solutions) S is found for $\mathbf{M} : 1 \times (k + 1)$, a solution is picked at random from S and a new row (generated at random from D) is added to $\mathbf{M}$, making it a $2 \times (k + 1)$ action matrix and so on. For initialization, $\bar{P}$ containing the probability of choosing rows in $\mathbf{M}$ is generated uniformly at random. For a $q \times (k + 1), q > 1$ action matrix, we assume that only the newly added q^{th} row and $\bar{P}$ need to be optimized because the remaining rows have been optimized for the target network in previous steps. In other words, this procedure of adding new rows to $\mathbf{M}$ implies that the previous rows contain information about how nodes selected actions when their choices were restricted and adding new rows will provide them with more choices. $\bar{P}$ allows nodes to choose among various rows, which may change when new rows are added and hence needs to be considered in the optimization framework. Though this procedure might restrict the search space for the action matrix, it suffices to provide some insights about the applicability and ability of the action-based approach to synthesize networks. As described in Section 5, each row of the action matrix reflects the mixed strategies used by nodes for making connections, and the aim is to find the minimum number of such rows that corresponds to the smallest parameter space capable of synthesizing the target network using ABNG. More rows are added to the action matrix until at least one of the following criteria are met:

[1] Pareto dominance is defined in the same way as Pareto optimality, the only difference being that $\mathbf{M}' \in S$, i.e. the new solution, is compared to the current set of potentially efficient solutions.

- The newly added q^{th} row has probability close to zero in the converged solutions, i.e. $\bar{P}_q \approx 0$. This implies that the network structure can be explained equally well using a smaller action matrix. A threshold value of 0.05 is used in the present implementation.
- The newly added q^{th} row is similar to one of the previous rows i.e., $\mathbf{M}(q,:) \sim \mathbf{M}(b,:), 1 \leq b \leq q-1$. Similarity between two rows can be defined using any vector similarity measure. This implies that the two strategies are practically equivalent. In case such an event occurs during optimization, the $\bar{P}$ values for both the rows are added (and assigned to the initial row) and the copy row is deleted i.e. $\mathbf{M}(q,:)$ is deleted and $\bar{P}_b = \bar{P}_b + \bar{P}_q$.
- Solutions from a smaller action matrix strictly dominate the new solutions, i.e. $S(\mathbf{M}_{b\times(k+1)}) \succ S(\mathbf{M}_{q\times(k+1)})$, $1 \leq b \leq q-1$. This means adding new rows does not improve the quality of the synthesized networks for the given set of actions.

The modified version of Pareto Simulated Annealing used here also incorporates an adaptable number of maximum iterations (Algorithm S3 lines 16-20). After every 100 iterations, the previous 100 solutions are checked for improvement in any of the objectives. If $S_z \neq S_{z-100}$, 100 more iterations are allowed, otherwise the process is terminated at a maximum of 1000 iterations. The strategy resulted from the peculiar behavior of the optimization process that can be observed in Figure S7, where convergence of solutions is observed before reaching the maximum number of iterations. This adaptable approach aids the algorithm in identifying potential local optima and consequently stopping the optimization process.

S6.3 The Action Set

An action for a node v_i builds edges, all of which have v_i as one endpoint. An action provides a well-defined strategy for selecting the other end v_j of edge (v_i, v_j). This implies that the actions in ABNG can only allow local topological changes in the network as a node is assumed to only build edges to other nodes but cannot create edges between other nodes. Every action for node v_i returns another node v_j with probability $\hat{p}_i^j$ to form edge (v_i, v_j). So, for network $\mathcal{G} = \{V, E\}$:

$$A(V|i) : v_i \rightarrow (v_i, v_j) \quad \text{w.p.} \quad \hat{p}_i^j, \tag{S3}$$

where an edge (v_i, v_j) is inserted into $\mathcal{G}$ by v_i. This approach also enables an action (and hence ABNG) to insert edges that can be directed, weighted, self edges, etc. In this paper, it is assumed that actions are limited to adding a single undirected edge. Actions for removing, rewiring, or adding multiple edges can be easily added to the framework while following the guidelines given below:

- Adding, removing, rewiring edges should be based on some criteria rather than being random. They can be inspired from some real-world phenomena for forming connections or based on some common criteria for application to particular domains.

- A node should only have the ability to change its local structure.
- The action should reveal some information about the network construction mechanism in the target network.

This raises the question: *How many actions should be considered for synthesis of a network?* There is no direct answer for such a question, however, having too many actions will increase the number of parameters (size of the action matrix) and hence make solving the optimization problem more difficult and time-consuming. This might even lead to degenerate actions and consequently complicate the generator.

Actions form an integral part of ABNG. They collectively serve as the mechanisms responsible for network synthesis. Hence, choosing a holistic or sufficient set of actions is crucial for implementing ABNG. The current implementation of ABNG uses eight actions belonging to four different categories:

- **Preferential attachment using network centrality measures** - This is analogous to the Barabási-Albert Model [5], which uses node degree as an action to connect to different nodes. It connects to *important* nodes with higher probability, where importance is calculated using network centrality measures. We use degree (PAD), average neighbor degree (PAND), PageRank (PAPR) and betweenness (PAB) as centrality measures for four different actions. An important property of this set of actions is that $\hat{p}$ is the same for all the nodes.
- **Triadic closure** - This action (TC) connects a node to another node that is a neighbor of its neighbor. It captures the phenomenon: *a friend of my friend is also my friend.* Also, triangles form a commonly encountered structure in real-world networks [18]. In case a node has multiple second neighbors, each one has an equal probability of getting selected.
- **Similarity-based actions** - These provide a basis for nodes to connect to similar nodes, another phenomenon observed in most real-world networks [18]. Inverse log-weighted (SLW) and Jaccard similarity (SJ) measures are used in this implementation. Nodes connect to the most similar unconnected node for preventing multiple edges.
- There is another action (NA) that does not connect the current node to any other node. As we can see from Algorithm S1 , ABNG visits every node in the network to form new edges, and this action exempts a node v_i from making a connection, i.e. $\hat{p}_i = 0$.

S7 Results

S7.1 Comparing multiple Rows

Figure S2 compares results obtained from optimized 1-row, 2-row and 3-row action matrices for the forest fire model and the real-world network of co-appearances of characters in Victor Hugo's novel "Les Miserables".

Network Name	n	m	z	l	c	r
Erdös-Rényi	100	500	10	2.236	0.103	0.041
Power Law	100	500	10	2.225	0.165	-0.089
Small World	100	500	10	2.383	0.275	-0.085
Barabási-Albert	100	485	10	2.208	0.177	-0.102
Forest Fire	100	311	6.22	3.089	0.361	-0.071
Stochastic Block	100	430	8.6	2.700	0.140	0.27
Word Adjacencies	112	425	7.59	2.535	0.157	-0.129
Political Books	105	441	8.4	3.078	0.348	-0.128
Co-appearances	77	254	6.59	2.641	0.499	-0.165
Jazz Collaborations	199	2742	27.56	2.235	0.520	0.02
Football Games	115	616	10.71	2.508	0.407	0.142
Network of Dolphins	62	159	5.13	3.357	0.309	-0.044
Brain (cor=0.7)	129	327	5.07	7.57	0.512	0.552
Brain (cor=0.6)	239	1039	8.69	4.87	0.542	0.577
Brain (cor=0.55)	252	1499	11.89	3.98	0.557	0.574
Biogrid FRET	987	1747	3.54	6.76	0.013	0.40
Biogrid Far Western	622	1073	3.45	5.27	0.010	-0.12
Biogrid Dosage Lethality	994	1780	3.58	3.41	0.002	-0.36
Protein 1php	394	1256	6.38	6.34	0.17	0.286
Protein 1qop	655	2243	6.85	6.82	0.193	0.35
Yeast Protein	426	521	2.45	6.02	0.021	-0.195
US Airports	500	2980	11.92	2.99	0.351	-0.268
Norwegian Boards (Aug. 2011)	854	2745	6.43	6.66	0.624	0.052
Human Protein	4100	13358	6.52	4.06	0.033	-0.216
Social Network	1893	13835	14.62	3.06	0.057	-0.188
US Power Grid	4941	6594	2.669	18.989	0.103	0.003

Table S2: List of target networks along with some network properties: total number of vertices n; total number of edges m; mean degree z; mean vertex–vertex distance l; clustering coefficient c; and degree correlation coefficient r.

Adding a second row to the action matrix improved the solution quality, but adding a third row did not improve the quality of the synthesized networks with respect to the objectives taken into account here. In other words, the 2-row solutions Pareto dominated the 1-row solutions, while the 3-row solutions lead to a $Q(\mathcal{G}|Y, \mathbf{M})$ that was equivalent to the 2-row solutions and hence showed no improvement. Also, the 3-row solutions had $\bar{P}_2 \approx 0$, which implies that there were only two distinct *nodes types*.

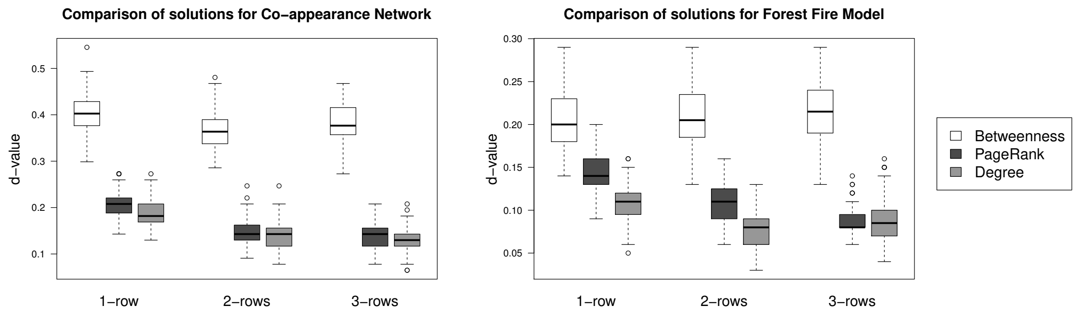

Figure S2: Improvement using 2-rows: This box plot highlights the effect of adding additional rows to the action matrix. For the networks shown here, adding a second row to the action matrix produced solutions that Pareto dominated the solutions from a 1-row action matrix. Each box plot shows statistics from 100 networks generated using ABNG.

S7.2 ABNG for Real Networks

19 real-world networks were also considered and details are shown in the table. Real-world networks will likely not be simply described like the human-devised models in the previous experiments. Radar plots for the networks synthesized using action matrices obtained as solutions for these real-world networks can be seen in Figure S3. The estimated action matrices can potentially provide insights about the structure of these networks like, how many types of nodes exist in the network, how they weigh actions to form edges etc. Description of the action-based model for five of these networks can be seen in Section 3. Figure S3 also shows statistics when some other network generators, namely Chung-Lu [9, 8] and ERGM [2], were used to fit the target network. From the comparison it can be concluded that:

- Networks synthesized using ABNG show the most resemblance to the target networks when evaluated based on the network properties considered here.
- Unlike other models, the output parameters obtained using ABNG (action matrices) can provide a compact representation of structure of the target networks.

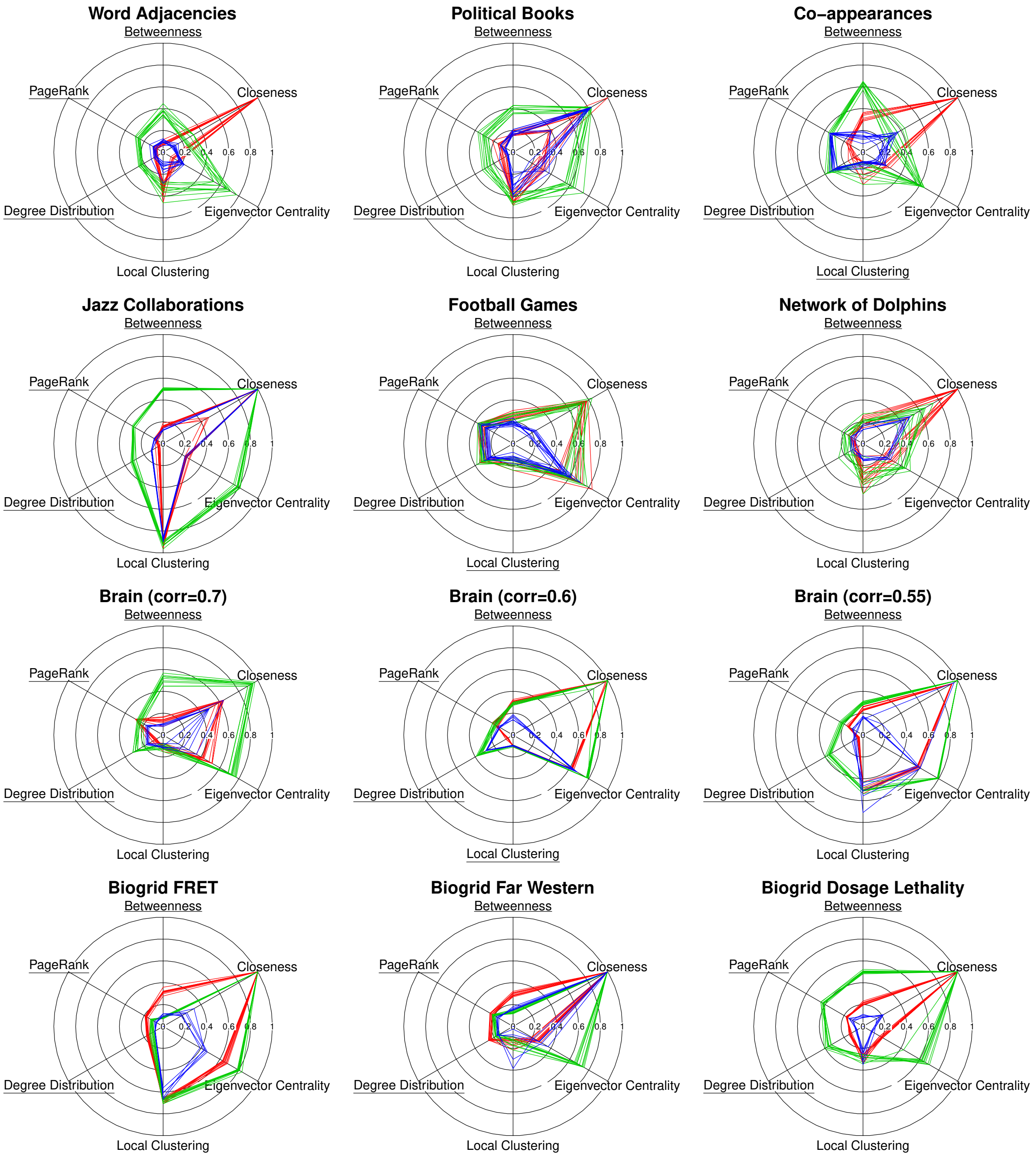

Figure S3: Results obtained from the optimized ABNG models for the real-world networks considered here. Network properties that were used for optimization are underlined in the radar plot. The plots show KS-test *d-statistic* with the outer circle showing value of 1 (maximum possible value). The lower the value, better is the synthesized network. Figure continues on next page.

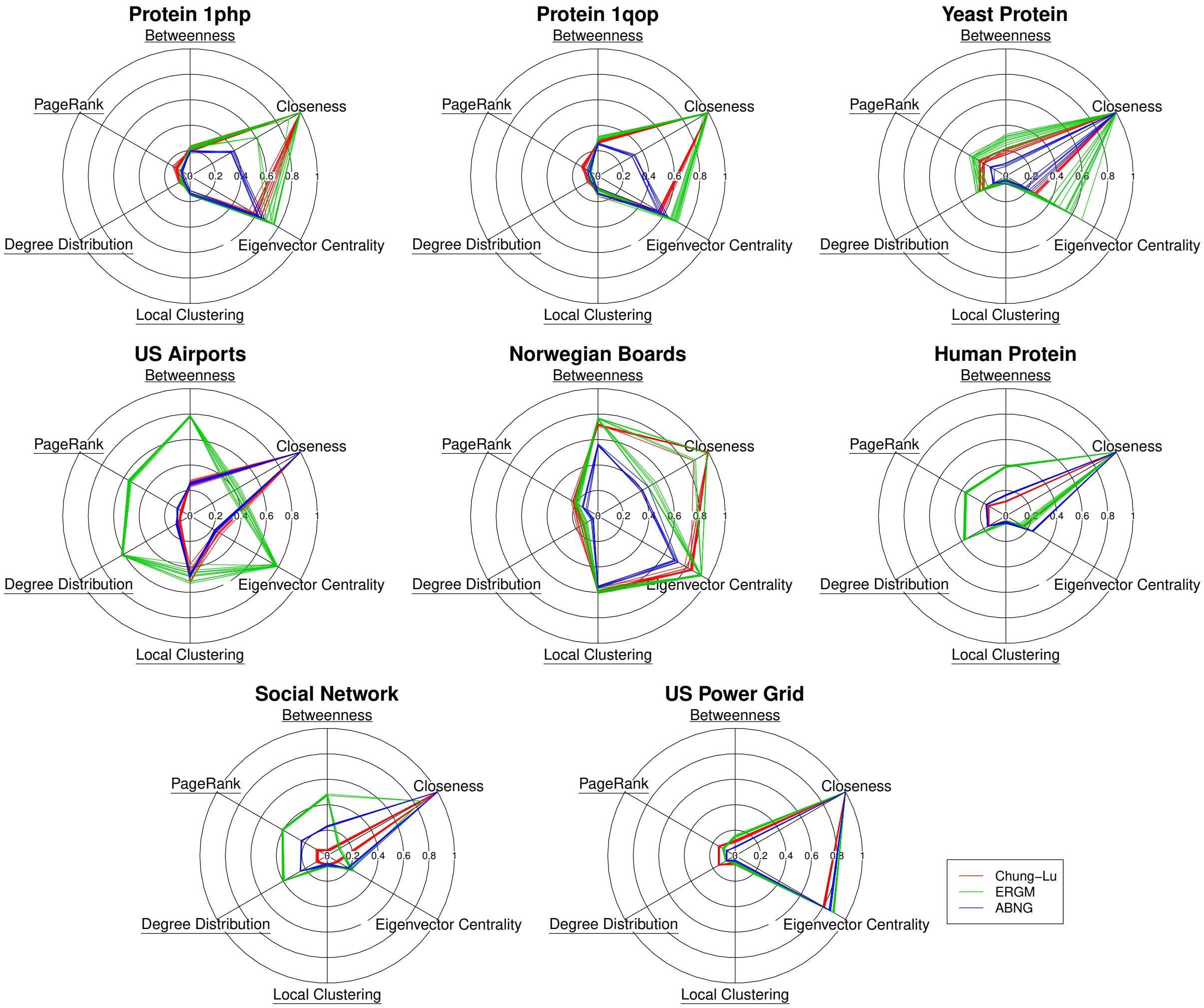

S7.3 Scaling with Network Size

An experiment was also performed to get empirical insights about scalability of the synthesis algorithm described in Algorithm S1. As described in Section S3, the complexity of any given synthesis algorithm depends on the input action matrix together with the size of the network. To understand the relation between network synthesis time and size of the network (number of nodes), an experiment was performed where each trial used a different action matrix and the size of the network was increased. Also, the mean degree of the networks was kept constant ($\bar{d} = 6$) as the number of nodes in the network was varied. Mean degree of 6 was chosen based on observations of mean degree of real-world networks shown in Table S2. Results shown in Figure S4 provide preliminary insights that network synthesis time scales quadratically with number of nodes in the network. The fitted quadratic model predicts that a network with 100,000 nodes and $\bar{d} = 6$ will require around 1 hour for synthesis. For network of each size, 20 networks were synthesized parallely using ABNG-PA(1) and the total CPU time was recorded. The plot shows mean times for each network size and action matrix. The system used consisted of 10-core Intel Xeon-E5 CPUs with a

frequency of 2.60GHz.

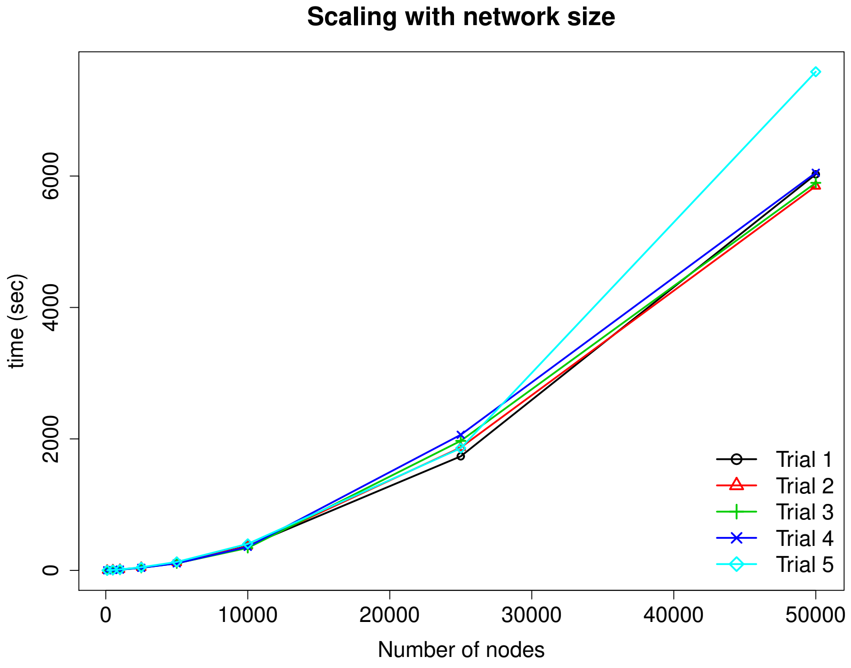

Figure S4: Scaling of ABNG-PA(1) with network size: Five different action matrices were used as input for networks containing different number of nodes. All networks have a mean degree of 6 and networks with 100, 500, 1000, 2500, 5000, 10000, 25000 and 50000 nodes were used.

S7.4 Starting Network Variations

In the experiments conducted so far, it is assumed that the starting network is obtained by sampling $0.7 \times n$ edges from the target network. In this section, different proportion of links are sampled from the target network to see how the generator performs when provided with different starting network. For this, we consider four different target networks and five different fraction of links, as shown in Figure S5. The synthesized networks are evaluated based on five network measures and corresponding heat maps are shown in Figure S5. For each target network, the solution closest to the origin was chosen as the action-based model to synthesize 20 networks and average values for each measure are recorded in the heat maps. The heat maps show that the quality of the synthesized networks does not depend much on the fraction of links in the starting network. Only when the fraction of links in the starting network is $0.25 * n$, a consistent drop in quality is observed for each target network.

In earlier experiments, a fixed starting network was used for synthesis throughout the optimization process (see Figure 1). While varying the fraction of links in the starting network, we also relaxed the assumption of using a fixed starting network and sampled a different starting network each time the algorithm was used

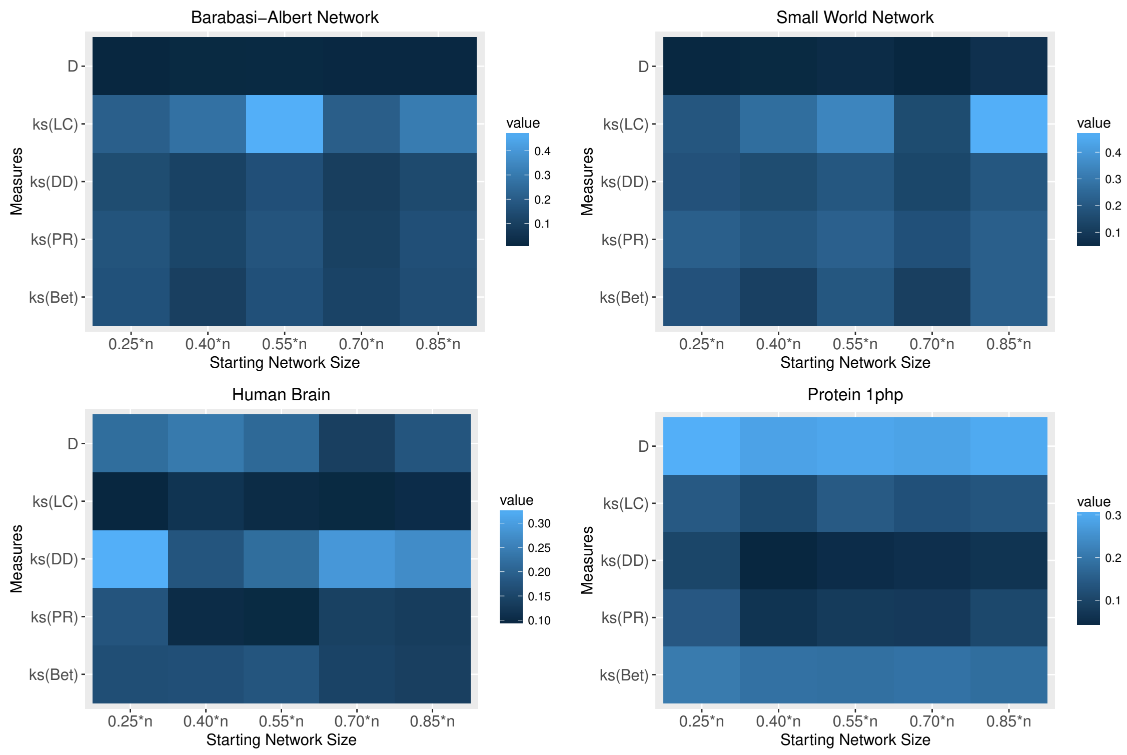

Figure S5: Starting network variation: Each generator synthesizes 20 networks, and the mean dissimilarity values for different measures are recorded in the heat maps. The lower the value, better is the synthesized network.

to synthesize a network. Results indicate that ABNG can synthesize networks having similar Q when the assumption of a fixed starting network is relaxed, hence providing evidence about the robustness of the action-based approach.

S7.5 Spectral Goodness of Fit

A new statistic to evaluate how well a network generator explains the structure of the pattern of ties in the target network was proposed in [25]. The current version of the *Spectral Goodness of Fit* (SGOF) statistic is limited to only unlabeled and undirected networks, which matches with the type of networks synthesized using ABNG. Because of its simplicity, we use SGOF as a proxy measure for goodness of fit for the synthesized networks and do not consider it in the optimization process of ABNG.

The approach calculates the Euclidean Spectral Distance ($E\bar{S}D_{\mathcal{T},\mathcal{G}} = ||\hat{\lambda}^{\mathcal{T}} - \hat{\lambda}^{\mathcal{G}}||$), where $\hat{\lambda}^{\mathcal{T}}$ and $\hat{\lambda}^{\mathcal{G}}$ are the normalized spectra (of the Laplacian) of networks $\mathcal{T}$ and $\mathcal{G}$. The spectral goodness of fit (SGOF) can be then obtained by:

$$SGOF = 1 - \frac{E\bar{S}D_{\mathcal{T},\mathcal{G}}}{E\bar{S}D_{\mathcal{T},\mathcal{N}}} \tag{S4}$$

where $\mathcal{N}$ is the null model. For SGOF calculations, the Erdös-Rėnyi model is used as the null model. The

SGOF measures the amount of observed structure ($\mathcal{T}$) explained by a fitted model ($\{\mathcal{G}_1, \mathcal{G}_2, \cdots\}$), expressed as a percent improvement over a null model, where structure means deviation from randomness [25].

SGOF is bounded above by one, which means that the network generator (or fitted model) exactly describes the target network. Similarly, an SGOF of zero means that synthesized networks are only as good as the random networks, whereas a negative value signifies that the null model is a better approximation of the target network as compared to networks synthesized using the network generator. Figures S6a and S6b show the SGOF values obtained from 100 networks synthesized using different network generators for both human-devised and real-world networks. The networks for ABNG are synthesized using the action matrix corresponding to the point closest (based on 1-norm distance) to the origin in the Pareto front. Note that SGOF values close to zero are observed for the Erdös-Rényi network because it is itself used as the null model.

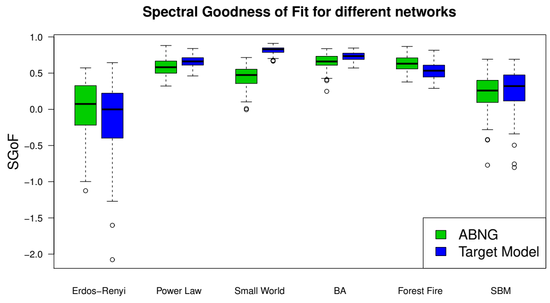

(a)

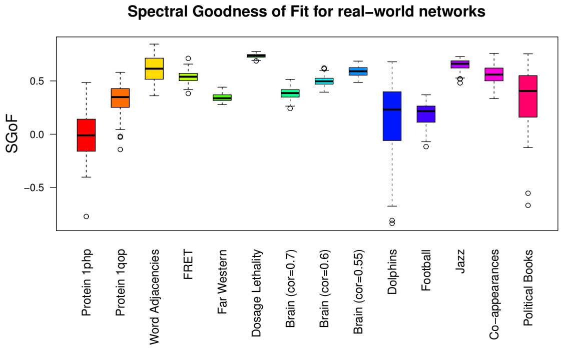

(b)

Figure S6: S6a Spectral Goodness of Fit for human-devised generators: The plot compares SGOF values for networks synthesized using ABNG and the target model. Each box plot corresponds to SGOF values obtained from comparing 100 synthesized networks with the target network. It can be seen that for most cases ABNG performs as well as the target model. S6b Spectral Goodness of Fit for real-world networks: SGOF values obtained for different real-world networks synthesized using ABNG. Each box plot corresponds to SGOF values obtained from comparing 100 synthesized networks with the target network.

To maintain consistency of using a dissimilarity metric ranging between 0 and 1, we transform the obtained SGOF value by using the function $y = 1 - 2^{x-1}$, where y gives us the transformed dissimilarity and x is the SGOF value obtained using S4. This transformation is used in the heat maps of Table 2.

S7.6 Analyzing the Action Matrix

Here, the evolution of the action matrix and the objectives during the PSA iterations are examined to get a better understanding of the process of learning an action matrix in ABNG. Figure S7 illustrates two examples representative of typical evolution of solutions for 1-row and 2-row action matrices when using PSA for optimization in ABNG. The optimization process shows typical characteristics observed in evolutionary

multi-objective optimization algorithms, where a lot of improvement is seen in the objective space in the first few iterations and the solutions seem to converge in the later iterations. Also, it can be seen that the best solutions are found before reaching the maximum number of iterations and can be observed when the curves become flat in Figure S7.

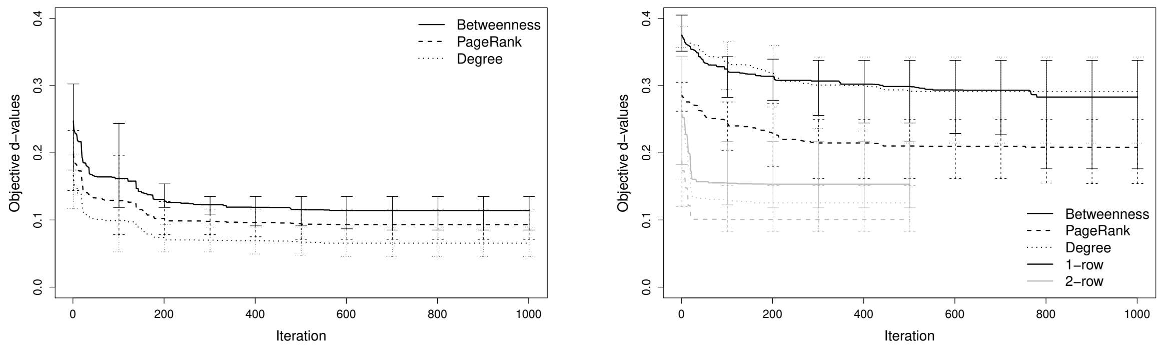

(a) (b)

Figure S7: Iteration plots for ABNG action matrix optimization using PSA: The plots show evolution of specific objectives versus the number of PSA iterations. Each line depicts averaged results over 5 restarts for PSA. Error bars show the minimum and maximum values obtained in different iterations of PSA. S7a is for a 1-row action matrix. S7b shows plots for both a 1-row limit and 2-row limit of the action matrix for a network synthesized using the forest fire model.

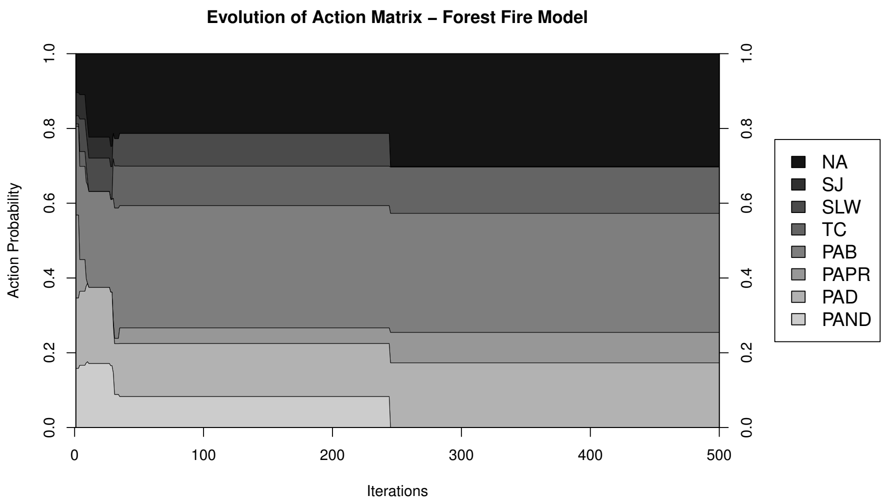

Figure S8: Example action matrix evolution: This shows the evolution of an action matrix versus number of iterations of PSA for a network synthesized using the forest fire model.

Examining different solutions obtained for the same target network leads to an interesting observation. Table S3 shows the cosine similarity of 5 different Pareto optimal action matrices ($\mathbf{M}_1 - \mathbf{M}_5$) for the

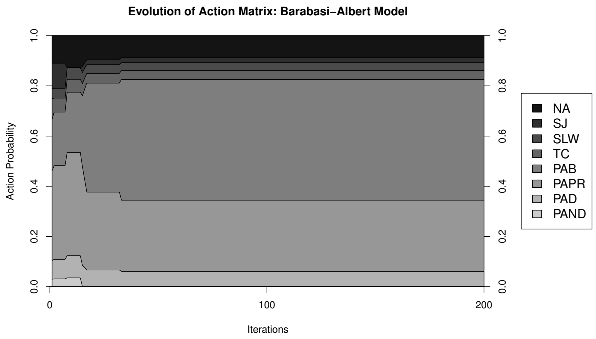

(a)

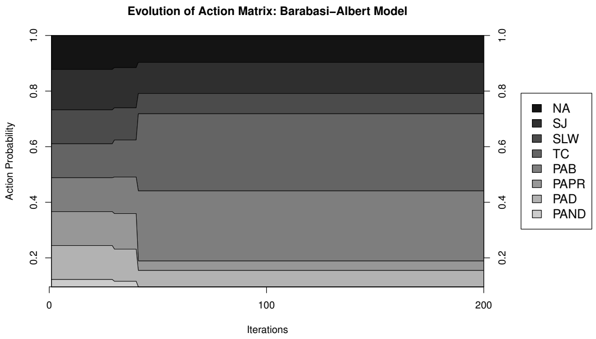

(b)

Figure S9: Action Matrix evolution: This shows the evolution of an action matrix versus number of iterations of PSA for the Barabási–Albert model. S9a Starting with a random action matrix. S9b Starting with uniform probability distribution over actions.

Barabási–Albert model. As is evident, the solutions are very similar because of the high cosine similarity values (the difference is likely to be because of the granularity of the optimization approach), and hence ABNG identifies similar underlying mechanism of network formation each time it optimizes the action matrix for a target network. It shows that the ABNG algorithm consistently finds the same solutions for a target network. Figures S8 and S9 show different examples for the evolution of a 1×8 action matrix. Figure S9a shows a plot when the optimization process starts with a randomly generated starting point, while Figure S9b shows the evolution of action matrix when the starting solution had equal probability for each action. Similar to Figure S7, the solutions stabilize after certain number of iterations. Also, some actions have very high probability in the final solutions while others might have zero probability. Though the results shown in Figures S7-S9 are for a few particular networks, they are representative of the other networks that have been considered in this paper and hence capture the behavior of ABNG.

Action Matrix	$\mathbf{M}_1$	$\mathbf{M}_2$	$\mathbf{M}_3$	$\mathbf{M}_4$	$\mathbf{M}_5$
$\mathbf{M}_1$	1.000	0.996	0.976	0.982	0.985
$\mathbf{M}_2$	0.996	1.000	0.988	0.992	0.996
$\mathbf{M}_3$	0.976	0.988	1.000	0.999	0.993
$\mathbf{M}_4$	0.982	0.992	0.999	1.000	0.997
$\mathbf{M}_5$	0.985	0.996	0.993	0.997	1.000

Table S3: The table shows cosine similarity of five different optimal solutions for the Barabási–Albert model.

To visualize the diversity of the solutions based on the size of the action matrix, a 3D plot (Figure S10) is shown for the three objectives used in the optimization process for the brain network with correlation threshold of 0.7. The example is representative of the distribution of Pareto optimal points obtained when ABNG is optimized for a target network. It is observed that the solutions are more spread out (or scattered)

for smaller action matrices (1-row and 2-row) and the solutions seem to be more concentrated at a particular region when considering larger action matrices.

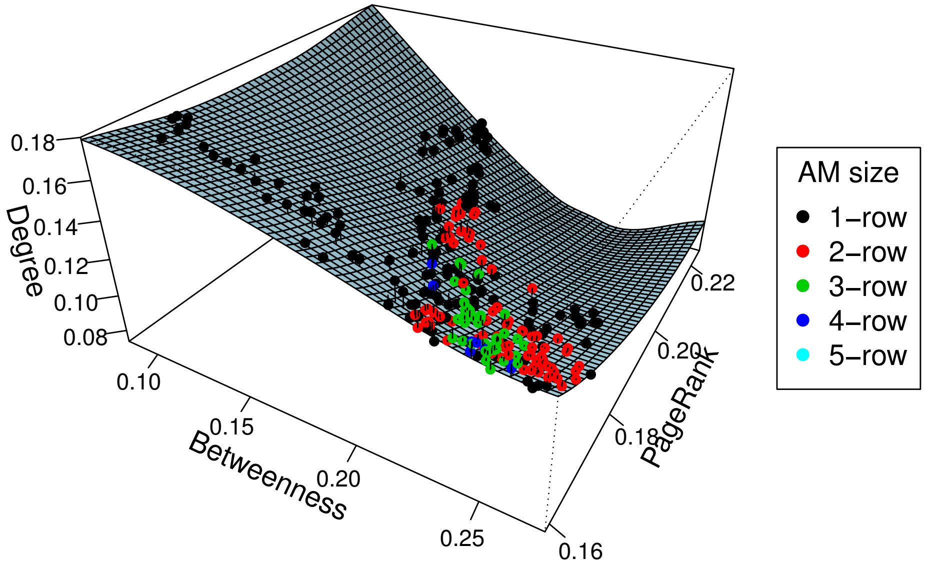

Figure S10: A 3D plot is presented to characterize the Pareto front obtained by ABNG optimization process. The plot shows how different solutions (in terms of action matrix size) are spread out in the objective space.

S7.7 Sensitivity Analysis of the Action Matrix

Another set of experiments involved performing a sensitivity analysis of the action matrices obtained from the optimization process. This helps us understand how the uncertainty in the output of ABNG can be apportioned to different sources of uncertainty in its inputs ($\mathbf{M}$ and $\bar{P}$). Two types of analysis were done:

1. Change one-variable-at-a-time (OAT), i.e. independently changing the probability of each action by 10%. This captures change in the output due to a change in probability of using a single action and provides evidence for the stability of the outcome. Radar plots for the four different target networks with an optimized 1×8 action matrix are shown in Figure S11. It is clear that there is very little variation in the synthesized network properties especially for the properties considered in the optimization framework (underlined in the plot). This is evident from the radar plots as the lines corresponding to change in probability of different actions overlap each other.

   Sensitivity of the action matrix was also tested for a 2-row action matrix obtained for the forest fire model network. In this scenario, the same approach was used to separately perturb the first row, second row and $\bar{P}$ associated with the action matrix. Again, the radar plots of Figure S12 provide evidence for the stability of the outcome even in the case of a bigger action matrix.

2. The one-at-a-time (OAT) approach does not fully explore the input space since it does not take into account the simultaneous variation of input variables. This means that the OAT approach cannot detect

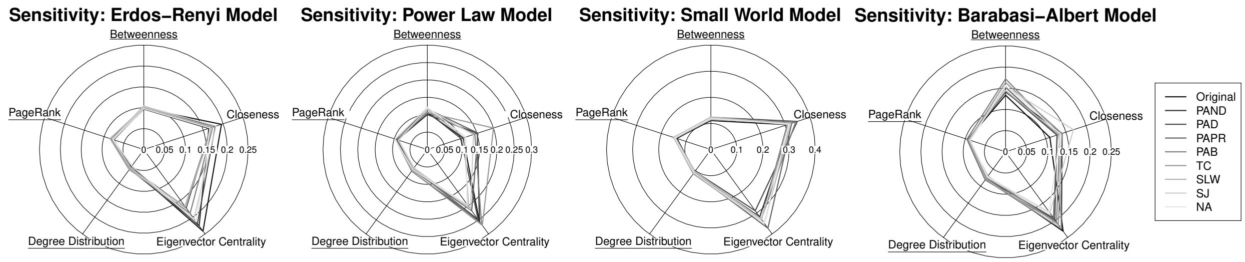

Figure S11: Sensitivity Analysis: The plots show KS-test *d-statistic* for different network properties. Network properties that were used as objectives for optimization are underlined. Each line corresponds to average of 20 networks synthesized using a 1-row action matrix perturbed using OAT approach.

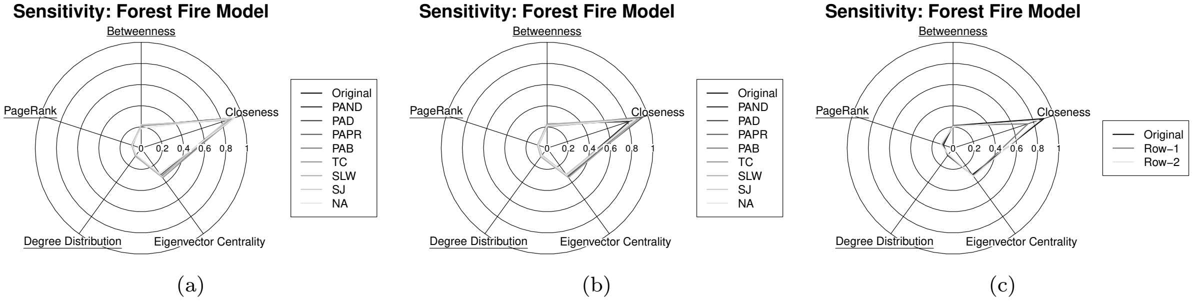

Figure S12: Sensitivity Analysis: The plots show KS-test *d-statistic* for different network properties. Network properties that were used as objectives for optimization are underlined. Each line corresponds to average of 20 networks synthesized using a OAT perturbed action matrix where S12a is for the first row, S12b is for the second row and S12c is for $\bar{P}$.

the presence of interactions between input variables. Instead, the second test varies the probabilities of all the actions simultaneously. Radar plots for this version of the sensitivity analysis can be seen in Figure S13 and S14 for the 1-row and 2-rows cases respectively. For each network, the test is performed five times with distinct variations in the action matrix. Clearly, even using this method there is a lot of overlap in the synthesized network properties for the different variations of the action matrix, especially for the properties considered as objectives for the optimization. The same is true for the case of a 2-row action matrix considered in Figure S14.

Results for both the cases reflected the robustness of the solutions obtained by showing that making small perturbations to the action matrix had little effect on the output of the synthesized networks. This provides preliminary evidence for the continuity in mapping the action matrix to the objective space and that the quality of the synthesized target networks is robust to small changes in parameters.

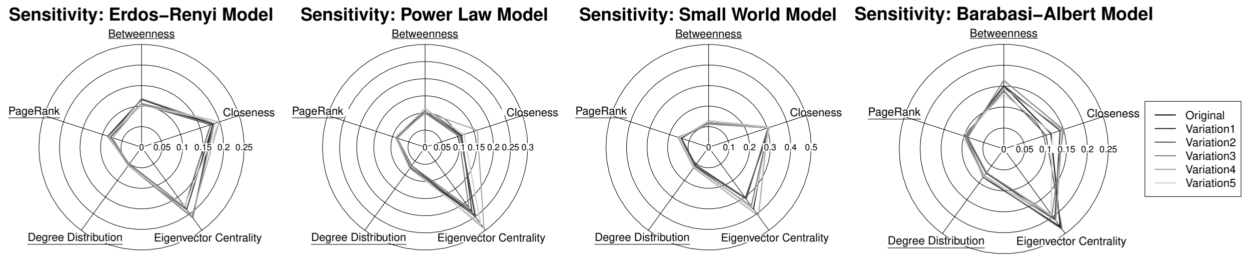

Figure S13: Sensitivity Analysis: The plots show KS-test *d-statistic* for different network properties. Network properties that were used as objectives for optimization are underlined. Each line corresponds to average of 20 networks synthesized using a simultaneously perturbed action matrix.

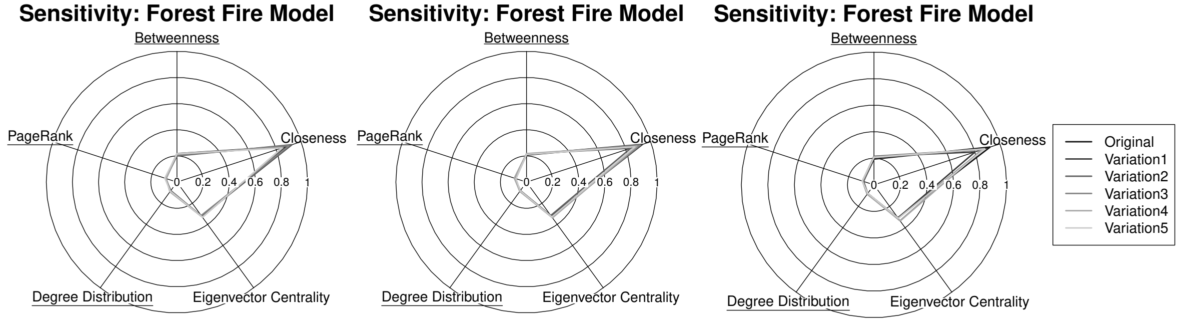

(a) (b) (c)

Figure S14: Sensitivity Analysis: The plots show KS-test *d-statistic* for different network properties. Network properties that were used as objectives for optimization are underlined. Each line corresponds to average of 20 networks synthesized using a simultaneously perturbed action matrix where S14a is for the first row, S14b is for the second row and S14c is for $\bar{P}$.

S7.8 Datasets and Packages

The implementation of Action-based network generator has been done using the "igraph" package in R. This involved synthesizing target networks from other human-devised network generators and performing statistical analysis on various networks. The "spectralGOF" package in R was used to compute SGOF values for different networks. Following real world networks were used: network of word adjacencies [19], US politics books sold on Amazon [1], a network of coappearances [16], network of American football games [12], collaboration network between Jazz musicians [13], a social network of dolphins [17], brain networks (with different correlation cut-offs) [6], two protein networks [7], a network of yeast protein interactome [15], three networks obtained from the Biogrid repository [26], US Airport network [10], Norwegian boards network [23], human protein interaction network [22], social network from an online community for students at University of California, Irvine [20], and a network representation of the topology of the Western States Power Grid of the United States [27].

The protein networks 1php and 1qop were obtained from the Protein Data Bank [7]. The .pdb files

obtained from this database contains information about all atoms composing a given protein. This can be used to obtain contact maps containing key relations from the protein structure. The C-alpha atoms were chosen from the .pdb files and a contact map was obtained using a threshold of 8 Å, i.e. if the distance between two atoms i and j is less than 8 Å, then the undirected link (v_i, v_j) exists. The networks Biogrid FRET, Far Western and Dosage Lethality were obtained from the biogrid database available at [26]. Brain fMRI data was obtained from the NITRC KKI dataset available at The Neuro Bureau ADHD-200 Preprocessed Repository [6]. The parcellation scheme described in [24] was used to define nodes for network analysis. Correlation matrix for patient 1019436 was used at correlation cutoffs 0.7, 0.6 (brain 2), and 0.55 (brain 3) to generate the respective networks (largest component of the network was used as the target network). The Norwegian boards network [23] shows interaction among board members of public companies in Norway as obtained from the data in August 2011. The human protein interaction network was obtained from [22] and the largest connected component was used as the target network. The US Airport network [10] shows connections between airports (nodes) if there is a direct flight between them.

References

[1] L. A. Adamic and N. Glance. The political blogosphere and the 2004 US election: divided they blog. In *Proceedings of the 3rd international workshop on Link discovery*, pages 36–43. ACM, 2005.

[2] C. J. Anderson, S. Wasserman, and B. Crouch. A p* primer: logit models for social networks. *Social Networks*, 21(1):37–66, 1999.

[3] D. Asta and C. R. Shalizi. Geometric Network Comparison. nov 2014.

[4] A. Bailey, M. Ventresca, and B. Ombuki-Berman. Genetic Programming for the Automatic Inference of Graph Models for Complex Networks. *IEEE Transactions on Evolutionary Computation*, 18(3):405–419, 2014.

[5] A.-L. Barabasi and R. Albert. Emergence of scaling in random networks. *Science*, 286(5439):509–512, oct 1999.

[6] P. Bellec, C. Chu, F. Chouinard-Decorte, Y. Benhajali, D. S. Margulies, and C. R. Craddock. The Neuro Bureau ADHD-200 Preprocessed Repository. Technical report, jan 2016.

[7] H. M. Berman. The Protein Data Bank. *Nucleic Acids Research*, 28(1):235–242, jan 2000.

[8] F. Chung and L. Lu. Connected Components in Random Graphs with Given Expected Degree Sequences. *Annals of Combinatorics*, 6(2):125–145, 2002.

[9] F. Chung and L. Lu. The average distances in random graphs with given expected degrees. *Proceedings of the National Academy of Sciences of the United States of America*, 99(25):15879–15882, dec 2002.

[10] V. Colizza, R. Pastor-Satorras, and A. Vespignani. Reaction–diffusion processes and metapopulation models in heterogeneous networks. *Nature Physics*, 3(4):276–282, apr 2007.

[11] P. Czyzak and A. Jaszkiewicz. Pareto Simulated Annealing–A Metaheuristic Technique for Multiple-Objective Combinatorial Optimization. *Journal of Multi-Criteria Decision Analysis*, 7(1):34–47, 1998.

[12] M. Girvan and M. E. J. Newman. Community structure in social and biological networks. *Proceedings of the National Academy of Sciences*, 99(12):7821–7826, 2002.

[13] P. M. Gleiser and L. Danon. Community Structure in Jazz. *Advances in Complex Systems*, 6(4):565–573, 2003.

[14] K. R. Harrison, M. Ventresca, and B. Ombuki-Berman. Investigating Fitness Measures for the Automatic Construction of Graph Models. In A. M. Mora and G. Squillero, editors, *EvoApplications*, volume 9028 of *Lecture Notes in Computer Science*, pages 189–200. Springer, 2015.

[15] T. Ito, T. Chiba, R. Ozawa, M. Yoshida, M. Hattori, and Y. Sakaki. A comprehensive two-hybrid analysis to explore the yeast protein interactome. *Proceedings of the National Academy of Sciences*, 98(8):4569–4574, apr 2001.

[16] D. E. Knuth. *The Stanford GraphBase : A platform for combinatorial computing.* Boston, 2009.

[17] D. Lusseau, K. Schneider, O. J. Boisseau, P. Haase, E. Slooten, and S. M. Dawson. The bottlenose dolphin community of Doubtful Sound features a large proportion of long-lasting associations. *Behavioral Ecology and Sociobiology*, 54(4):396–405, 2003.

[18] M. Newman. *Networks: An Introduction.* Oxford University Press, 2010.

[19] M. E. J. Newman. Finding community structure in networks using the eigenvectors of matrices. *Physical review E*, 74(3):36104, 2006.

[20] T. Opsahl and P. Panzarasa. Clustering in weighted networks. *Social Networks*, 31(2):155–163, 2009.

[21] C. Orsini, M. M. Dankulov, P. Colomer-de Simón, A. Jamakovic, P. Mahadevan, A. Vahdat, K. E. Bassler, Z. Toroczkai, M. Boguñá, G. Caldarelli, S. Fortunato, and D. Krioukov. Quantifying randomness in real networks. *Nature Communications*, 6(May):8627, 2015.

[22] T. Rolland, M. Taşan, B. Charloteaux, S. Pevzner, Q. Zhong, N. Sahni, S. Yi, I. Lemmens, C. Fontanillo, R. Mosca, A. Kamburov, S. Ghiassian, X. Yang, L. Ghamsari, D. Balcha, B. Begg, P. Braun, M. Brehme, M. Broly, A.-R. Carvunis, D. Convery-Zupan, R. Corominas, J. Coulombe-Huntington, E. Dann, M. Dreze, A. Dricot, C. Fan, E. Franzosa, F. Gebreab, B. Gutierrez, M. Hardy, M. Jin, S. Kang, R. Kiros, G. Lin, K. Luck, A. MacWilliams, J. Menche, R. Murray, A. Palagi, M. Poulin, X. Rambout, J. Rasla, P. Reichert, V. Romero, E. Ruyssinck, J. Sahalie, A. Scholz, A. Shah, A. Sharma, Y. Shen,

K. Spirohn, S. Tam, A. Tejeda, S. Trigg, J.-C. Twizere, K. Vega, J. Walsh, M. Cusick, Y. Xia, A.-L. Barabási, L. Iakoucheva, P. Aloy, J. De Las Rivas, J. Tavernier, M. Calderwood, D. Hill, T. Hao, F. Roth, and M. Vidal. A Proteome-Scale Map of the Human Interactome Network. *Cell*, 159(5):1212–1226, 2014.

[23] C. Seierstad and T. Opsahl. For the few not the many? The effects of affirmative action on presence, prominence, and social capital of women directors in Norway. *Scandinavian Journal of Management*, 27(1):44–54, 2011.

[24] X. Shen, F. Tokoglu, X. Papademetris, and R. Constable. Groupwise whole-brain parcellation from resting-state fMRI data for network node identification. *NeuroImage*, 82:403–415, nov 2013.

[25] J. Shore and B. Lubin. Spectral goodness of fit for network models. *Social Networks*, 43(0):16–27, oct 2015.

[26] C. Stark, B.-J. Breitkreutz, T. Reguly, L. Boucher, A. Breitkreutz, and M. Tyers. BioGRID: a general repository for interaction datasets. *Nucleic acids research*, 34(Database issue):D535–9, jan 2006.

[27] D. J. Watts and S. H. Strogatz. Collective dynamics of 'small-world' networks. *Nature*, (393):440–442, 1998.
